# Platelet Dynamics in Neurodegenerative Disorders: Investigating the Role of Platelets in Neurological Pathology

**DOI:** 10.3390/jcm13072102

**Published:** 2024-04-03

**Authors:** Maria Piera L. Cadoni, Donatella Coradduzza, Antonella Congiargiu, Stefania Sedda, Angelo Zinellu, Serenella Medici, Alessandra Matilde Nivoli, Ciriaco Carru

**Affiliations:** 1 Department of Biomedical Sciences, University of Sassari, 07100 Sassari, Italy; mariapieracadoni@libero.it (M.P.L.C.); a.congiargiu@phd.uniss.it (A.C.); s.sedda4@studenti.uniss.it (S.S.); azinellu@uniss.it (A.Z.); carru@uniss.it (C.C.); 2 Department of Chemical, Physical, Mathematical and Natural Sciences, University of Sassari, 07100 Sassari, Italy; sere@uniss.it; 3 Department of Medical, Surgical and Experimental Sciences, University of Sassari, 07100 Sassari, Italy; anivoli@uniss.it; 4 Psychiatric Unit Clinic of the University Hospital, 07100 Sassari, Italy

**Keywords:** platelet dynamics, neurological health, cognitive function, platelet activation, neuroinflammation

## Abstract

**Background:** Neurological disorders, particularly those associated with aging, pose significant challenges in early diagnosis and treatment. The identification of specific biomarkers, such as platelets (PLTs), has emerged as a promising strategy for early detection and intervention in neurological health. This systematic review aims to explore the intricate relationship between PLT dynamics and neurological health, focusing on their potential role in cognitive functions and the pathogenesis of cognitive disorders. **Methods:** Adhering to PRISMA guidelines, a comprehensive search strategy was employed in the PubMed and Scholar databases to identify studies on the role of PLTs in neurological disorders published from 2013 to 2023. The search criteria included studies focusing on PLTs as biomarkers in neurological disorders, their dynamics, and their potential in monitoring disease progression and therapy effectiveness. **Results:** The systematic review included 104 studies, revealing PLTs as crucial biomarkers in neurocognitive disorders, acting as inflammatory mediators. The findings suggest that PLTs share common features with altered neurons, which could be utilised for monitoring disease progression and evaluating the effectiveness of treatments. PLTs are identified as significant biomarkers for detecting neurological disorders in their early stages and understanding the pathological events leading to neuronal death. **Conclusions:** The systematic review underscores the critical role of PLTs in neurological disorders, highlighting their potential as biomarkers for the early detection and monitoring of disease progression. However, it also emphasises the need for further research to solidify the use of PLTs in neurological disorders, aiming to enhance early diagnosis and intervention strategies.

## 1. Introduction

Neurocognitive disorders constitute a broad category of mental health disorders that primarily affect cognitive functions, including memory, attention, perception, problem-solving, and language [1]. The onset can be ascribed to various factors, including neurological damage, brain injury, genetic factors, or underlying medical conditions, significantly impairing an individual’s ability to think, remember, learn, and carry out daily activities [2].

Although Alzheimer’s disease (AD) accounts for the majority of neurocognitive cases, other disorders are also included in this category, such as frontotemporal degeneration (FTD), vascular dementia (VD), Huntington’s disease (HD), traumatic brain injury (TBI), Parkinson’s disease (PD), delirium, and mild cognitive impairment (MCI) [3].

Neurodegenerative disorders are significantly associated with an aging population [4]. As people age, they undergo cellular changes that can contribute to the development of these conditions [5]. For instance, certain cells become less efficient and accumulate waste products, leading to inflammation and damage [6]. Over time, these damaged cells can die off, causing further harm to the brain [3,7]. Moreover, aging also affects the immune system, which plays a crucial role in preventing neurodegeneration [8]. As we age, the immune system becomes less effective at clearing away abnormal proteins that can build up and accumulate in the brain, contributing to the development of neurodegenerative diseases [9].

However, it is important to note that, while aging is a major factor in the increased prevalence of neurodegenerative diseases, it does not guarantee that an individual will develop one of these conditions [4]. Many older individuals remain healthy and free of these diseases throughout their lives. Other factors, including genetic predisposition, environmental influences, overall health status, and lifestyle factors such as diet, exercise, and stress levels, also play critical roles in determining whether an individual will develop such a condition [10].

The increase in the global aging population brings about rising healthcare expenditures due to the prevalence of neurodegenerative disorders [11]. Healthcare expenditures are substantial and include costs for diagnosis, treatment, caregiving, and long-term care facilities [12,13].

The main challenge is to reach early diagnosis through the identification of specific biomarkers that can help perform the early identification of vulnerable individuals and intervene with effective treatments. 

This necessity has led researchers to shift their focus from the central nervous system (CNS) to other body systems, such as peripheral blood, in the study of neurodegenerative disorders [14]. Although neurocognitive conditions have traditionally been identified as pathologies affecting the neuronal sphere, with all studies and diagnoses focused on the CNS, the past few years have seen a shift from this neuro-centric vision [15,16]. Evidence revealed that the characteristic hallmarks of pathologies affecting neuronal cells can be identified in both neurons and peripheric cells [17,18,19,20,21] and that also other systems, outside the CNS, take part in the pathogenic process of neurocognitive disorders. Furthermore, neuroinflammation [22,23,24,25] as well as environmental factors and thus microorganisms and viruses, have been involved in the pathogenesis of neurodegenerative disorders [26,27]. Therefore, the peripheral system has become a new field of study for various neurological pathologies, providing relevant data in both sera and peripheral cells [3]. The observation of pathologic hallmarks in peripheral cells opened a new era in the research for diagnostic and prognostic markers [28,29]. CNS is a difficult seat on which to conduct a study, while peripheral blood is an easily accessible source through no invasive method and it is a useful tool to study and perform the diagnosis of these pathologies [30]. However, it remains to be clarified whether peripheric alterations only reflect the damage present in the central nervous system or are a part of the pathologic process [31]. 

Among all the biomarkers studied, platelets (PLTs) showed a surprising role and involvement in the pathogenesis of cognitive disorders [32]. 

PLTs are fragmented cells derived from megakaryocytes. Human PLTs present a diameter that ranges from 1 to 5 μm, depending on their physiological functions as well as their number, usually comprising between 150.000 and 400.000/μL of blood [33]. 

Although anucleate, PLTs are equipped with complete cytoplasm, including mitochondria, messenger ribonucleic acid (mRNA), and secretory vesicles, such as lysosomes, dense granules, and α-granules, and are able to maintain and process nucleic acids based on environmental influences [34,35,36]. PLTs are usually associated with their role in the haemostatic process, whereby they initiate clotting following endothelial disruption or tissue injury [37,38]. However, the rich content of mediators present within their granules, which are released upon specific stimuli, as well as the large number of receptors identified on their surface, reveal that these fragmented cells participate in numerous systemic pathways that extend far beyond the coagulation process [39]. Leiter and Walker in their review effectively summarised all the functions ascribed to PLTs, such as abilities in tissue remodelling, brain plasticity, neurotrophic factors secretion, synaptic plasticity, exosome secretion and inflammation, and an involvement in cross talk between central nervous system and periphery [34]. All these findings show that PLTs are important in homeostatic maintenance and also reveal their possible implication in neuropathological disorders when their functions are altered [40]. 

In this work, through a systematic review of the last ten years, we aimed to investigate the role of PLTs in neurocognitive disorders paying particular attention to AD, FTD, VD, HD, TBI, PD, delirium, and MCI, to deepen the role that these fragmented cells play and in which way their alteration can be used as biomarkers as well as the therapeutic target.

## 2. Materials and Methods

### 2.1. Search Strategy and Selection Criteria

Following the PRISMA guidelines and adhering to the Grades of Recommendation, Assessment, Development, and Evaluation (GRADE) criteria [41,42,43], this systematic review was conducted from 10 December 2023 to 10 January 2024. An extensive search was carried out across PubMed, Scopus, and Web of Science databases. The search strategy involved utilising keywords such as “Neurodegenerative diseases”, “Aging”, “Cellular changes”, “Inflammation”, “Damage”, “Immune system”, “Abnormal proteins”, “Lifestyle factors”, “Genetic predisposition”, “Environmental influences”, “Health status”, “Biomarkers”, “Platelets (PLTs)”, “Cognitive disorders”, “Neuroinflammation”, “Peripheral blood”, “Pathologic process”, “Systematic review”, “Alzheimer’s disease (AD)”, “Frontotemporal degeneration (FTD)”, “Vascular dementia (VD)”, “Huntington’s disease (HD)”, “Traumatic brain injury (TBI)”, “Parkinson’s disease (PD)”, “Delirium”, and “Mild cognitive impairment (MCI)” to precisely define the scope of the literature. The inclusion criteria involved the exploration and reporting of the aforementioned factors in patients. Literature was excluded if it fell into categories such as review articles, pre-2013 publications, or non-English language articles. Additional relevant material was sought by screening the reference lists of the identified literature and previous review articles. The exclusion criteria were carefully applied, encompassing materials such as abstracts, letters, reviews, case reports, etc. Studies with insufficient data for comprehensive analysis were omitted. In cases where multiple publications originated from the same cohort, only the most recent report was included for our meta-analysis.

### 2.2. Data Collection and Quality Assessment

The literature satisfying our eligibility criteria and incorporated into this review underwent data extraction by the authors. The key data points comprised details of the study (author and date), study design, total number of patients, their diagnoses, specific outcomes measured, and study results. The assessment of the literature incorporated in this review aligned with the GRADE criteria, which assesses the quality of evidence and provides recommendations for use [41,44,45]. These criteria encompass the quality of the methodology, directness of evidence, heterogeneity, precision of effect estimates, and the potential for publication bias. This resulted in assigning a level of evidence and recommendation for use, categorised as high, moderate, or low.

## 3. Results and Discussion

### 3.1. Literature Search and Study Characteristics

**Figure 1:**
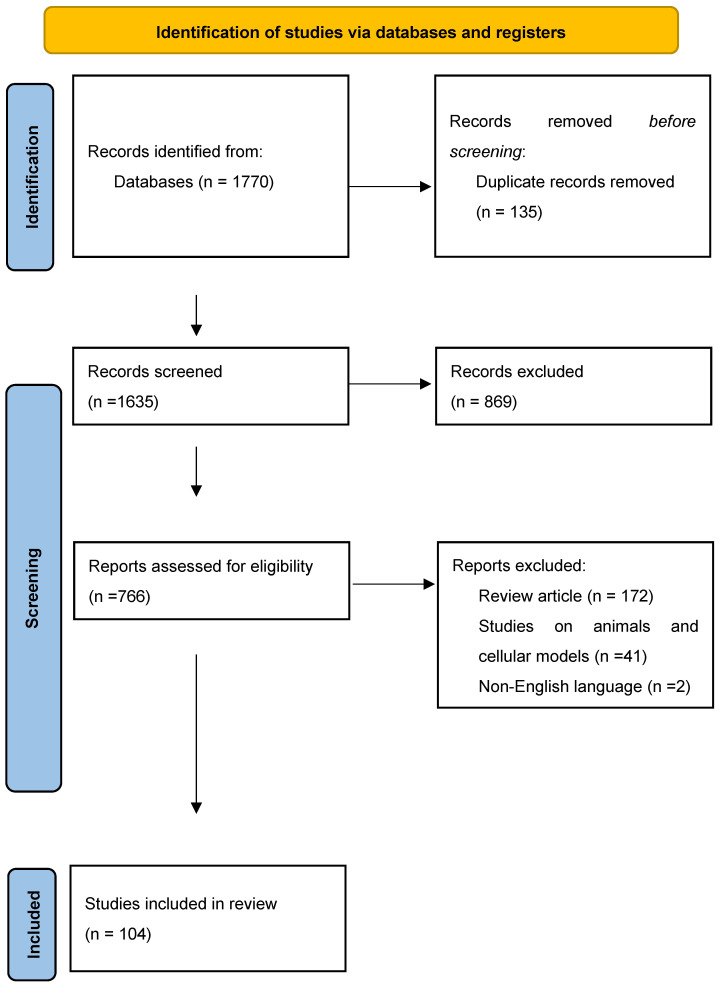
PRISMA flow diagram [46] representing the literature search and study selection process.

The implementation of the search strategy yielded a total of 1770 records initially identified as relevant to the research question. After removing duplicate records in EndNoteX9, the titles and abstracts of 1635 records were screened against the inclusion and exclusion criteria. At this stage, 869 records were excluded, leaving 766 records for the full-text assessment of eligibility. Among these, 215 articles were excluded for being review articles, studies conducted on animal or cellular models, or published in a non-English language. Consequently, 104 full-text articles were deemed relevant and included in the qualitative and quantitative analysis of this systematic review, as reported in Figure 1.

### 3.2. Summary of Platelet–Neurocognitive Disorder Relationships

This systematic review aimed to investigate the intricate role of PLTs in neurocognitive disorders paying particular attention to AD, FTD, VD, HD, TBI, PD, delirium, and MCI. All these pathologies are connected by a common thread, represented by cognitive decline that can be ascribed to various conditions, such as the accumulation of misfolded proteins (AD and PD) [47,48,49], triplet expansion (HD) [50], or to other medical conditions such as what happens in delirium [51,52]. In this expansive landscape, increasing evidence supports the strong relationship between neurocognitive disorder and vascular systems [53,54] in which PLTs seem to play a role in triggering inflammatory processes by releasing mediators that in turn attract from blood circulation other inflammatory cells which, by accumulating in the blood–brain barrier, concur to alter its integrity [34]. This evidences how the CNS, considered for long time an immune privileged seat, is subjected to the immune system action, which contributes to neuronal damage triggering neuroinflammatory processes. In addition, it is widely demonstrated that PLTs share molecular alterations common to those reported in neurons, such as accumulation and the release of mutant proteins [55,56,57], the alteration of lipid homeostasis, the alteration of mitochondrial function, oxidative stress, and so on [58,59,60]. All these findings lead to consider PLTs as the closest peripheral neuronal-like cellular system useful to study neurological disorders [61]. In this view, PLTs appear to have a dual role: (i) as the orchestrator in the complex mechanisms that lead to brain damage and thus, neuronal loss and memory deficits [61,62,63] and (ii) as peripheral biomarkers, that can be used to monitor disease progression and therapeutic efficacy. All these functions will be described below for each pathology.

### 3.3. Unlocking the Role of Platelets in Alzheimer’s Disease

Alzheimer’s disease (AD) is a complex and multifactorial neurodegenerative disorder, representing the most common type of dementia [62]. Currently, approximately 47 million people are affected by dementia, a number projected to reach 131 million by 2050 [32]. A distinctive feature of AD in the brain is that of cortical atrophy, predominantly in the medial temporal lobe, while at the microscopic level, damaged tissues exhibit the combined presence of extracellular amyloid plaques, composed of beta-amyloid peptides (Aβ), and intraneuronal neurofibrillary tangles, composed of tau protein. Both of these components consist of highly insoluble and densely packed filaments [64]. Recent evidence emphasises that pathologies affecting the neuronal sphere, including AD, result from a pathological process in which not only neuronal cells but also the nervous system participate in the pathogenesis and progression of the disease [25,32,63,65]. In this regard, various studies conducted on blood samples have revealed the involvement of peripheral cells, including PLTs, which seem to share a biochemical alteration typical of neurons in AD, such as increased β-secretase activity and the amyloidogenic processing of the amyloid precursor protein. They also appear to play a role in the pathological process of AD [61]. It has been widely demonstrated that PLTs contain high levels of Amyloid Precursor Protein (APP), and that both APP and Aβ are stored in the α-granules of PLTs, released upon their activation [66]. Researchers have extensively studied the presence of APP and Aβ in PLTs to gain diagnostic and prognostic insights into AD. In [19], it was demonstrated that the levels of Aβ42, a toxic peptide derived from APP, showed a positive correlation with the platelet count in AD patients. Furthermore, these levels were associated with disturbed mitochondrial respiration in the PLTs of individuals with AD [67]. These findings emphasise the potential of platelet-related parameters as markers for AD progression. The exploration of APP in PLTs has extended to comparative analyses among AD patients, individuals with mild cognitive impairment (MCI, an early stage of AD), and healthy controls. This comprehensive examination has yielded intriguing data that could serve as foundational elements for developing diagnostic processes. Jelic et al., utilising a monoclonal antibody capable of detecting the N-terminal part of AβPP, identified two distinct AβPP patterns: 130 kDa and 105 kDa species, already documented in the literature, and a novel 115 kDa AβPP form. Significantly, the 115 kDa AβPP species exhibited a notable increase in the PLTs of individuals with mild cognitive impairment and AD compared to control subjects. Notably, they discovered a correlation between the abundance of the 115 kDa AβPP species and Mini-Mental State Examination scores [57]. This correlation suggests that, with further investigation, the 115 kDa AβPP species could potentially serve as a diagnostic marker for preclinical stages of AD. This line of research holds promise for advancing diagnostic approaches in the early detection and monitoring of AD.

Another study directed its research towards examining mRNA expression, specifically focusing on the total APP and the Kunitz-type serine protease inhibitor domain of APP (APP KPI) in PLTs. The study involved participants from various groups, including control subjects, individuals affected by AD, and those with Frontotemporal Lobar Dementia (FTLD) [68].

The findings from their investigation revealed a noteworthy upregulation of both the total APP and APP KPI in the PLTs of patients with both AD and FTLD when compared to control subjects. This differential gene expression suggests a potential role of these APP components in the pathophysiology of both neurodegenerative conditions. Furthermore, the study delved into the correlation between APP mRNA expression levels and cognitive impairment. Intriguingly, a statistically significant positive correlation was only observed in AD patients. This emphasises the potential utility of assessing APP mRNA expression levels in PLTs as a biomarker specifically associated with cognitive impairment in the context of AD. 

This study contributes valuable insights into the molecular mechanisms underlying neurodegenerative disorders. The APP amyloid precursor protein ratio 120–130/110 kDa fragments (APPr) in PLTs has been a widely scrutinised parameter, with a majority of studies conducted in the Caucasian population. These studies consistently reveal a significant decrease in APPr in PLTs derived from AD patients and those with MCI when compared to healthy subjects [69]. Srisawat et al. conducted a similar study in an Asian population, specifically among Taiwanese individuals [70]. Interestingly, their findings showed a statistically insignificant decrease in APPr in AD patients compared to the control group. This led the researchers to conclude that APPr might have limited utility in this particular cohort of patients, highlighting potential ethnic or regional variations in the relevance of this parameter. Beyond its role in discriminating AD patients from healthy subjects, APPr has been investigated for its potential in monitoring the effectiveness of cognitive training protocols [71]. The data obtained from this study are noteworthy, suggesting that APPr could serve as a valuable tool for assessing the efficacy of cognitive interventions. Furthermore, the findings raise the intriguing possibility that APPr might also be employed to monitor the effectiveness of drug treatments, offering a novel avenue for evaluating therapeutic outcomes in the context of neurodegenerative disorders. Marksteiner and their research group utilised APP to develop and validate an assay for secreted amyloid-precursor protein (sAPP)-α and -β in PLTs obtained from subjects with AD and MCI, comparing them to both healthy young and old controls [56]. The study revealed that sAPP-β levels in individuals with MCI and AD were significantly elevated in comparison to those in the control groups. This investigation underscores the potential of sAPP-β as a biomarker, providing valuable insights into the pathophysiological changes associated with neurodegenerative conditions. The elevation of sAPP-β levels in MCI and AD subjects suggests its potential utility in distinguishing individuals with cognitive impairment from healthy controls. Such assays contribute to the ongoing efforts in developing diagnostic tools and markers that can aid in the early detection and differentiation of neurodegenerative disorders. Evidence suggests that altered APP processing occurs before clinical manifestations in AD patients with mutations associated with the autosomal dominant form of the disease [72]. This observation highlights the potential of APP isoforms in PLTs as surrogate biomarkers for assessing disease progression and treatment effectiveness. In pursuit of this objective, a study conducted an evaluation of the TDP-43 profile in PLTs. Their findings mirrored those observed in the study of APP, suggesting a correlation between TDP-43 alterations and the pathological processes associated with AD [73]. This parallelism in results strengthens the hypothesis that APP isoforms in PLTs could indeed serve as valuable indicators for monitoring disease progression and treatment response. The exploration of TDP-43 in PLTs adds another layer of complexity to the understanding of molecular changes in neurodegenerative disorders. The convergence of findings with APP studies further supports the potential utility of PLT-based assessments in offering insights into the early stages of AD. 

APP undergoes proteolytic processing by three enzymes: α-secretase, β-secretase, and γ-secretase. Beta-site APP-cleaving enzyme 1 (BACE1) functions as the β-secretase, while A-disintegrin and metalloprotease 10 (ADAM10), a member of the ADAM family, is associated with α-secretase activity. Additionally, presenilin-1 (PSEN1) is a component of the γ-secretase complex. Investigations into these enzymes in PLTs obtained from AD patients and control groups aim to explore their potential as biomarkers for AD [74].

A study reported a statistically significant decrease in ADAM10 (52.5%, *p* < 0.0001) and PSEN1 (32%, *p* = 0.02) levels in PLTs from AD patients compared to controls. They further highlighted that a combination of ADAM10, BACE1, and PSEN1 protein levels demonstrated good accuracy in discriminating AD from controls [43]. However, these findings are in contrast to other studies [75]. Decourt et al. reported a significant reduction in BACE1 levels in AD compared to healthy controls [76], and in a separate study, the mRNA levels of ADAM10 were not significantly different among AD, MCI, and healthy controls [77]. Despite conflicting results, there are consistent data correlating ADAM10 levels with cognitive tests, indicating its potential use as a biomarker to discriminate AD from controls [78,79].

These discrepancies in findings may arise from variations in sample populations, methodologies, or disease stages, emphasising the need for further research and standardisation in assessing these enzymes as potential biomarkers for AD.

It is crucial to acknowledge that the isolation of PLTs, sample size, and various treatments can potentially influence the data obtained. Therefore, larger and more comprehensive studies are essential to ensure the reliability and generalisability of findings in the context of AD. 

While the pathology of APP and Aβ appears to be well established, the role of tau protein in AD remains poorly understood. Sarg et al. demonstrated that the tau protein is expressed in human PLTs in very low concentrations and primarily in a fragmented form. They observed a reduction in tau protein in AD patients compared to healthy subjects, emphasising the high heterogeneity of this protein, which may limit its use as a biomarker for AD [66]. In a separate study, another research group investigated tau protein and identified two forms: High-Molecular-Weight (HMW) and Low-Molecular-Weight (LMW) tau. They found an increase in HMW tau, while the second form, LMW tau, decreased. Additionally, they calculated the tau ratio between these two oligomers, suggesting that the HMW/LMW tau ratio could serve as a valuable biomarker for AD. This ratio was associated with measures of disease severity and pathology, indicating its potential as an indicator for the progression of AD [20]. These findings highlight the complexity and heterogeneity of tau protein in PLTs. 

In recent years, inflammation has been identified as a crucial process contributing to AD pathogenesis. PLTs have emerged as active participants in this inflammatory process, leading to investigations into various PLTs parameters. One research group utilised atomic force microscopy to characterise PLTs, identifying specific characteristics that allowed for the differentiation of patients affected by AD, amyotrophic lateral sclerosis, and Parkinson’s disease [80]. A similar study conducted by Wiest and their group focused on the comparison of PLTs’ characteristics between individuals with AD and normal subjects [81]. On the contrary, Tirozzi et al. did not observe any significant correlation between the PLTs’ parameters and the risk of developing AD [82]. However, Koc et al. reported a significant increase in Mean Platelet Volume (MPV) in AD patients compared to the controls [83]. Notably, using MPV, they also found differences between the moderate and mild Mini-Mental State Examination (MMSE) groups, suggesting a potential association between MPV and cognitive decline in AD. Additionally, MPV was found to be higher in Aβ-positive patients compared to Aβ-negative controls [84], aligning with previous studies indicating an increased MPV in AD. These findings collectively underscore the intricate relationship between PLT parameters and AD, highlighting certain characteristics, especially MPV, as potential indicators of disease presence, severity, and cognitive decline. 

PLT parameters serve as a reflection of their activity, and the identification of alterations in these parameters underscores their potential role in AD. However, it is important to note that further investigations are warranted, considering that PLT activity can be influenced by various drugs and other conditions that may induce inflammatory responses. 

In addition to traditional PLTs parameters, researchers have explored the expression of adenosine A2A, an endogenous neuromodulator known to regulate synaptic plasticity. This neuromodulator has been implicated in memory deficits associated with AD. The elevation of A2A in the brains of AD patients is a well-established finding. Merighi et al. extended this understanding by demonstrating that PLTs from AD patients exhibit the same alteration in A2A expression [85]. This connection between A2A dysregulation in both the brain and PLTs highlights the potential of PLTs as a peripheral model to study the aspects of AD pathology. 

The investigation into neuromodulators in PLTs provides insights not only into the systemic changes associated with AD, but also into the potential mechanisms underlying cognitive deficits. 

A broad spectrum of PLT parameters has been investigated in AD, revealing a correlation between neuronal and PLT characteristics. In addition to the previously discussed data focusing on APP, tau, and Aβ, alterations in lipid metabolism, known to be disrupted in neuropathological diseases, including AD, have been observed in PLTs [55,86]. These lipid alterations exhibit specific differences that can aid in distinguishing individuals with AD from normal controls. 

Furthermore, a study highlighted a correlation between the specific variants of *apolipoprotein E (APOE)* and mitochondrial activity by studying cytochrome oxidase [87]. *APOE* is a well-known genetic risk factor for AD, and this study suggests a link between *APOE* variants and mitochondrial function in PLTs. Moreover, the APOE4 variant in PLTs have also been correlated with the acceleration of cerebrovascular injury and cognitive decline [88].

Understanding these relationships may provide valuable insights into the mechanisms underlying AD pathogenesis and the potential role of PLTs in reflecting systemic alterations associated with the disease. 

The exploration of lipid metabolism and mitochondrial activity in PLTs contributes to the comprehensive understanding of the peripheral manifestations of AD. These investigations not only aid in distinguishing AD from normal controls but also offer potential avenues for identifying biomarkers and therapeutic targets. 

In conclusion, based on research conducted over the last decade, PLTs emerge as an intriguing source for the pathological signature of AD. Through miRNA analysis [89,90], proteomic assays [91,92], and the evaluation of serotonin (5-HT) uptake [32,93,94], a diverse array of potential AD biomarkers has been identified [95,96]. Additionally, other studies indicate that PLTs may reflect apoptotic events occurring in neurons [97]. 

The literature suggests that PLTs serve as a reliable and non-invasive tool that mirrors neuropathological alterations present in neurons. The findings highlight the potential of PLTs to be representative of the molecular changes associated with AD. Future studies could benefit from more specific comparisons, not only between AD and healthy subjects but also including patients who share pathological signatures with AD. This approach may aid in the identification of specific biomolecular markers crucial for early diagnosis. One intriguing question that arises from the literature is whether the modifications observed in PLTs are a consequence of AD or whether PLTs play a more direct role in the neuropathology of the disease. The answer to this question holds potential implications for our understanding of the disease mechanisms and may open new avenues for therapeutic interventions. Further research is warranted to unravel the intricate relationship between PLTs and AD pathophysiology and to explore the possibility that PLTs may act as active players in the development and progression of AD (Table 1 and Table 2).

### 3.4. Role of Platelets in Parkinson’s Disease Pathogenesis and Biomarker Discovery

Parkinson’s Disease (PD) presents a challenging landscape in neurodegenerative research, and recent investigations highlight PLTs as pivotal players in its intricate pathogenesis. This chapter delves into the multifaceted interactions between PLTs and PD, elucidating their contributions to inflammation, neuroinflammation, potential biomarker discovery, and cognitive decline. Through a comprehensive exploration, this chapter aims to provide a nuanced understanding of the nuanced relationships between PLTs and PD [98].

PLTs are orchestrators of inflammation and neuroinflammation. PLTs, conventionally recognised for their haemostatic roles, emerge as key contributors to inflammatory processes in PD [53]. Upon activation, PLTs unleash a cascade of inflammatory molecules, thereby intensifying systemic inflammation. In the context of neurodegenerative diseases like PD, this heightened inflammation becomes a driving force for neuronal damage, subsequently propelling disease progression [99]. Furthermore, PLTs, upon activation, release neurotrophic factors crucial for neuronal survival and growth [100]. Yet, an intricate balance must be maintained, as an aberrant activation–deactivation equilibrium may result in an overproduction of neurotrophic factors, potentially contributing to neuronal death in PD [101]. Beyond this, PLTs also play a role in neural plasticity, a critical facet of learning and memory, through serotonin release [102]. This dual role of PLTs in both recovery from brain injury and potential contribution to neurodegeneration underscores their complexity in the context of PD [103].

Recent studies emphasise PLTs as promising sources for biomarker discovery in PD. The application of RNA sequencing on PLTs from PD patients and healthy controls has unveiled myriad differentially expressed genes. Among these, *MALAT1*, *EEF1A1*, and *CTSS* have been implicated in PD. Gene ontology analyses underscore inflammation-related pathways, suggesting that PLTs RNA profiling stands as a promising avenue for not only understanding PD’s pathogenesis but also unearthing potential biomarkers [104].

The utility of PLTs as cellular models for neurological diseases, given their similarities in 5-HT accumulation mechanisms with dopaminergic neurons, has become a key avenue of exploration. Studies involving freshly isolated blood PLTs from PD patients, control individuals, and those with Parkinsonism have unearthed intriguing findings. PD patients displayed a marked decrease in 5-HT content and uptake, along with compromised thrombin-induced release in PLTs. This impairment was notably absent in most Parkinsonism cases, highlighting a potential biomarker for PD. This defect in PLTs function not only hints at subclinical PD detection but also provides a platform for evaluating disease-modifying drugs [105].

In-depth investigations into platelet dysfunction in PD patients undergoing preoperative evaluation for deep brain stimulation have revealed abnormalities unrelated to medical history or drug use. Additionally, scrutinising PLTs mitochondrial membrane potential in PD patients has provided valuable insights. While the intact mitochondrial membrane potential in PLTs from PD patients indicates compensatory mechanisms, it also suggests potential subtypes of PD. This emphasises the imperative need to explore various cell types, including PLTs, for a comprehensive understanding of PD pathophysiology [106,107].

A novel therapeutic approach involves purified human PLTs lysate concentrated in neurotrophins as a potential treatment for PD. The enrichment of neurotrophins in this lysate raises the prospect of leveraging PLTs functions to address PD symptoms. However, the experimental nature of this strategy necessitates further research to fully comprehend the underlying mechanisms and evaluate its clinical effectiveness [108].

While exploring platelet-derived extracellular vesicles (PEVs) containing amyloid-beta 1–42 (Aβ1–42), a study found a profound link between an increased PEV-Aβ1–42/PEV ratio and cognitive decline in PD patients with dementia. This not only suggests a potential role for Aβ1–42-containing PEVs as biomarkers for PD dementia but also opens avenues for a deeper understanding of the intricate connections between PLTs and cognitive aspects of PD [109,110].

In conclusion, PLTs emerge as versatile orchestrators in the complex landscape of PD. From their pivotal roles in inflammation and neuroinflammation to their potential as biomarkers and indicators of cognitive decline, PLTs offer valuable insights into the multifaceted nature of PD [111,112]. The diverse array of studies presented underscores the necessity for continued research, unravelling the intricate mechanisms and potential therapeutic avenues involving PLTs in PD [113]. As we navigate the nuanced interplay between PLTs and PD, we inch closer to a more profound understanding that could pave the way for innovative therapeutic interventions (Table 3 and Table 4).

### 3.5. Architects, Sentinels, and Witnesses in Vascular Dementia Unveiled

In the broad field of neurodegenerative diseases, PLTs have become a focal point, revealing complex relationships between vascular dysfunction and vascular dementia (VaD). This review explores recent studies, detailing the intricate aspects of PLTs’ involvement in these disorders. It covers a range of topics from the regulation of growth factors to the identification of potential biomarkers and innovative therapeutic approaches. These studies collectively highlight the essential role of PLTs in understanding and combating neurodegeneration. Within the extensive landscape of neurodegenerative diseases, the role of PLTs has become a significant area of interest, intricately linking cerebral small-vessel disease (CSVD) and VaD [114].

Each study contributes to the complex puzzle, illuminating the multifaceted involvement of PLTs and their potential as indicators for understanding, diagnosing, and treating these severe conditions [40].

A meticulous investigation was undertaken into the interplay between vascular function, tau positron emission tomography (PET) imaging, and global cognition in the context of AD. This study not only unveiled a significant correlation between soluble platelet-derived growth factor b (sPDGFRb) and tau PET levels but also hinted at a paradigm shift in our approach to AD. Addressing vascular health alongside amyloid and tau levels emerged as a potentially more effective strategy for preserving cognitive function, sparking hope for future diagnostic and therapeutic advancements [115].

A study delved into the subtleties of PLTs’ indices as potential indicators for distinguishing between VaD and AD. Their meticulous exploration revealed a significantly lower MPV and platelet distribution width (PDW) in patients with VaD and AD, opening avenues for novel diagnostic strategies. The correlation between MPV and MMSE scores suggested not only a role in assessing cognitive status but also hinted at the potential of targeting PLTs’ activation for the treatment and prevention of these neurodegenerative diseases [116].

A study led by researchers examining frontal cortical capillary pericytes post mortem shed light on the pivotal role of PLTs in preserving neurovascular integrity. By emphasising the significance of PLTs in regulating the platelet-derived growth factor (PDGF) signalling pathway, the study underscored the intricate interplay between PLT function and PDGF signalling in maintaining the delicate balance of the neurovascular unit. These findings provide a foundation for future research into therapeutic targets for post-stroke dementia and AD [117].

While exploring the connections between diabetes-related vascular risk factors, it was noted that there exists a relationship between PLTs’ indices and neurodegeneration biomarkers in the context of healthy aging and AD. The study hinted at the potential involvement of PLTs in inflammation and neuroinflammation, offering insights into the development and progression of AD. Targeting PLTs function, as observed in diabetes-related conditions, emerged as a promising avenue for the prevention and treatment of vascular dementia, offering hope for future interventions [84].

A study delved into the field of dementia diagnostics, evaluating white blood cell and PLT counts as potential biomarkers. Although the PLT counts did not show statistically significant differences between dementia subtypes, this study recognised the evolving landscape of dementia diagnostics. Conducted using the Sysmex XN-10 Automated Haematology Analyzer, the research contributed to ongoing investigations of biomarkers in dementia diagnostics, highlighting the intricate nature of these conditions [118].

In a multinational effort, researchers investigated the release of amyloid peptides β1–40 and β1–42 by human PLTs in response to various stimuli. Their findings provided a potential link between PLT activation and amyloid-related diseases, particularly AD. The modulation of platelet-dependent Aβ peptide release emerged as a promising therapeutic target, offering insights into strategies aiming to reduce amyloid plaque deposition and slowing disease progression.

In the vast realm of neurodegenerative diseases, the enigmatic role of PLTs has emerged as a focal point, weaving a complex narrative in the context of AD, CSVD, and VaD. This narrative journey unfolds as we delve into a series of studies, each a piece in the intricate puzzle, shedding light on PLTs’ multifaceted involvement and their potential as gateways to understanding, diagnosing, and treating these devastating conditions.

A study clarified that PLTs significantly contribute to the aggregation of amyloid-β in cerebral vessels in the realm of cerebral amyloid angiopathy (CAA). Integrating insights from this study, PLTs take centre stage as orchestrators of fibrillar Aβ aggregates, whose dance is intricately choreographed by the signalling induced by integrin αIIbβ3 and the release of clusterin. This study invites us to consider PLTs as potential therapeutic targets, offering the perspective of intervening at the very foundations of CAA and AD. Antiplatelet therapy emerges as a potential strategy to mitigate the formation of fibrils, promising hope for targeted treatments [119].

In a study, a symphony of late-life blood pressure, brain oxygenation, and Aβ accumulation is composed, creating a tapestry that intertwines VaD and AD. Elevated blood pressure, a double-edged sword, seemingly aids brain oxygenation while laying the foundation for severe arteriolosclerosis and cerebral amyloid angiopathy. This study crafts a narrative where PLTs might serve as silent witnesses, responding to vascular alterations, potentially influencing cerebral perfusion, and contributing to the delicate dance of Aβ. As the late-life decline in blood pressure precedes dementia onset, this study suggests that understanding this interplay could hold the key to decoding the complexities of disease progression [120]. Another study narrates a tale of PLT indices and their intricate dance with cognitive and functional status in older hospitalised individuals. In the ever-evolving storyline of aging, PLTs, though not consistent markers, cast shadows on the potential associations between the altered PLT activity and the complexities of concurrent health conditions. This study underscores the challenges of translating these associations into clinical use, emphasising the multifaceted nature of diseases beyond dementia [121] (Table 5).

In a significant exploration, researchers delved into the association between PDGF and post-stroke outcomes. This study, involving 309 stroke patients over a five-year follow-up period, unveils intriguing findings. Elevated levels of PDGF-AB/BB are linked to a decreased risk of recurrent vascular events in stroke patients. However, this study does not find a direct association between PDGF levels and cognitive decline. PLTs, crucial for haemostasis and blood clot formation, emerge as key players in releasing PDGF, a protein intricately involved in regulating cell growth, division, and angiogenesis. The elevated PDGF levels observed in this study had previously been correlated with atherosclerosis and plaque instability. The results of this study propose the potential utility of PDGF-AB/BB as a prognostic marker for recurrent vascular events in stroke patients, offering a nonacute therapeutic target alongside conventional treatments. While these findings suggest a promising role for PDGF in predicting vascular events, this study wisely highlights the imperative need for further research to validate these associations and deepen our understanding of the underlying mechanisms. Establishing the therapeutic relevance of PDGF in stroke patients would necessitate additional investigations into its potential as a target for nonacute interventions, opening avenues for advancements in stroke management [122].

As we examine each study, PLTs are portrayed not just as cell components, but as central figures, providing opportunities for further investigation, comprehension, and potentially reshaping our understanding of neurodegenerative diseases (Table 6).

**Table 5:** Role of platelet as biomarkers in vascular dementia (VaD).

Reference	Disease	Samples/Animal Models	Study Objective	Results
Albrecht et al. (2020) [115]	Vascular disfunction	138 patients	Investigating correlation between vascular function, tau positron emission tomography (PET) imaging, and global cognition in the context of Alzheimer’s disease.	Significant correlation between soluble platelet-derived growth factor b (sPDGFRb) and tau PET levels.
Qing-Cheng Liang et al. (2014) [116]	VaD AD	150 VaD patients. 110 AD patients. 150 HCs	Examining the association between platelet indices and VaD and AD.	Lower MPV and PDW in patients with VaD and AD.
Palix et al. (2022) [84]	Diabetes AD	105 AD patients	Evaluating correlations between diabetes- related vascular risk factors, platelet indices, and neurodegeneration biomarkers.	Potential role of platelets in inflammation and neuroinflammation.
Bayat et al. (2020) [123]	VaD		Investigating the effects of the early and late administration of platelet-rich plasma (PRP) on learning-memory and hippocampal synaptic plasticity.	Early PRP administration significantly enhances synaptic plasticity and memory.
Schröder et al. (2022) [118]	Dementia	97 patients	Evaluation of white blood cell and platelet counts as potential biomarkers in the differential diagnostics of dementia.	No statistically significant differences between dementia subtypes regarding WBC and platelet count.
Donner et al. (2016) [119]	Cerebral amyloid angiopathy (CAA)		Investigating the contribution of platelets in the formation of vascular Aβ deposits.	Activated platelets directly contribute to CAA by promoting the formation of Aβ aggregates and Aβ, activating platelets, creating a feed-forward loop.
Pan et al. (2022) [124]	Dementia	131 patients 131 HCs	Examining connection between dysfunctional platelets and the contributor to AD development.	Anti-platelet therapy was associated with a lower risk of dementia.
Tayler et al. (2023) [120]	VaD, AD, mixed dementia	20 patients 75 patients 31 patients	Investigating the relationship between blood pressure, hypertensive status, and the development of dementia.	Complex relationship between late-life blood pressure, disease pathology and vascular function in dementia.
Socha et al. (2019) [114]	Cognitive performance	754 patients	Evaluating the relationship between platelet indices and cognitive and functional performance.	There was no significant correlation between cognitive performance and platelet indices
Narasimhalu et al. (2015) [122]	Stroke	309 patients	Examination of the association between PDGF AB-BB and post-stroke outcomes.	Higher levels of PDGF-AB/BB were independently associated with a lower risk of recurrent vascular events in stroke patients.

### 3.6. Platelets as Pivotal Players in the Tapestry of Frontotemporal Lobar Degeneration

Frontotemporal lobar degeneration (FTLD) stands as a multifaceted spectrum of disorders, characterised by diverse clinical and pathological manifestations predominantly impacting the frontal and anterior temporal lobes of the brain. Its distinction as the second most prevalent cause of dementia in younger individuals introduces a diagnostic conundrum due to its inherent heterogeneity. Syndromes within FTLD, such as behavioural variant frontotemporal dementia (bvFTD) and primary progressive aphasia, exhibit notable overlaps with motor neuron diseases, further complicating diagnostic endeavours. Diagnosis hinges on clinical features, specific protein aggregates in histopathology, or genetic mutations. Nevertheless, a pressing need for reliable biomarkers persists to enhance the accuracy of FTLD diagnosis.

Recent strides in neurodegenerative research have turned attention toward PLTs as potential contributors to the diagnostic landscape. PLTs, recognised for their involvement in myriad of physiological and pathological processes, emerge as a promising source of biomarkers due to their ability to traverse the blood–brain barrier. One intriguing avenue of exploration centres on the role of platelet-activating factor (PAF) in neurodegeneration. As a potent pro-inflammatory mediator, PAF is implicated in leukocyte adhesion, chemotaxis, and degranulation. Elevated PAF levels have been correlated with cognitive impairment in AD, unveiling its potential significance in neurodegenerative processes. Furthermore, PAF antagonists exhibit promise in modulating the degradation of amyloid-β42 in neurons, suggesting a potential role in regulating neurodegenerative pathways. Notably, PAF’s association with the regulation of cholesterol ester hydrolases and its higher levels in AD patients compared to controls extend the relevance of this mediator beyond AD, potentially into the pathophysiology of FTLD.

This study delves into the intricate interplay between PLTs, PAF, and neurodegenerative diseases, with a specific emphasis on FTLD. By shining a spotlight on PLTs’ involvement in inflammation, immune response, and neurodegeneration, the research underscores their potential relevance in the complex landscape of FTD.

In parallel, Sebastian Schröder and colleagues embark on a compelling exploration, aiming to unravel the potential of white blood cell (WBC) and PLTs count as biomarkers in dementia diagnostics. Their research, aptly titled “White Blood Cell and Platelet Counts Are Not Suitable as Biomarkers in the Differential Diagnostics of Dementia”, employs a comprehensive approach to assess the utility of these blood cell metrics in distinguishing various types of dementia. Despite the rigorous methodology, the findings conclusively demonstrate that WBC counts, platelet counts, and related metrics do not exhibit significant differences across diverse dementia types. This underscores the urgent need for more reliable and specific biomarkers, both for accurate diagnostic differentiation and targeted treatment strategies [118].

A pivotal breakthrough emerges with the development of the Simple Western assay, a cutting-edge automated capillary nano-immunoassay pioneered by Fourier et al. This innovative method focuses on quantifying the total TDP-43 in human PLTs samples, offering a significant leap forward in the quest for dependable FTLD biomarkers (Table 7). The assay demonstrates commendable quantitative performance in PLTs samples, showing the linearity of signals and a within-run variability of 5.7%, indicative of precision and reliability. Notably, TDP-43 protein profiles in PLT samples align with positive controls, suggesting that PLTs could serve as an appropriate sample matrix for studying TDP-43 in FTLD patients [125].

The successful quantification of TDP-43 signals in PLT samples through the Simple Western assay opens new vistas in biomarker discovery for FTLD and potentially other neurodegenerative diseases. While this automated capillary nano-immunoassay holds promise as a reliable and accurate diagnostic tool, the researchers judiciously call for further confirmation studies on larger cohorts of patients to assess its diagnostic performance in clinical settings.

In conclusion, the combined findings from the exploration of PLTs, PAF, and the innovative Simple Western assay in the context of FTLD present a paradigm shift in our understanding of the disease. These pioneering studies not only shed light on potential biomarkers and therapeutic targets but also emphasise the critical need for continued research to unravel the intricate mechanisms underlying FTLD. The path ahead holds the promise of refining diagnostics, offering targeted treatments, and ultimately enhancing our ability to navigate the complexities of frontotemporal lobar degeneration (Table 8).

### 3.7. Platelet Dysfunction in Traumatic Brain Injuries

Traumatic brain injuries (TBIs) can induce PLT inhibition, a condition where the blood’s clotting ability becomes compromised, potentially resulting in heightened bleeding and life-threatening complications [126,127]. Recent research has uncovered a promising avenue for intervention in such cases—PLT transfusions have demonstrated efficacy in reducing the necessity for neurosurgical procedures and lowering mortality rates [128].

PLTs, vital for the healing process following a TBI, are prone to dysfunction, acting as an early indicator for TBI-induced coagulopathy [129]. While no direct link between PLT dysfunction and dementia has been established, TBIs are recognised precursors to various neurological conditions, including dementia. The involvement of PLT dysfunction in blood clotting may indirectly contribute to dementia. Excessive bleeding or complications arising from PLT dysfunction have the potential to inflict damage on the brain, consequently contributing to cognitive decline [130].

In severe cases of TBI, patients often exhibit PLT dysfunction, characterised by a diminished PLT count and an extended bleeding time. Furthermore, individuals with TBI typically display significantly reduced PLT responses to arachidonic acid (AA), a substance crucial for triggering platelet aggregation, in comparison to their healthy counterparts. This diminished response to AA is correlated with an elevated risk of bleeding complications [131].

Although a direct link between PLT dysfunction and dementia remains elusive, the recognised association between TBI and neurological conditions prompts the consideration of the potential indirect role of PLT dysfunction in dementia development. The impact of PLT dysfunction on excessive bleeding or related complications may contribute to cognitive decline in TBI patients [132].

Moreover, an intriguing study titled “Human Platelet Lysate Biotherapy for Traumatic Brain Injury: Preclinical Assessment” revealed promising prospects. This research suggests that human PLT lysate (HPL) biotherapy holds the potential to enhance recovery from TBI by fostering neurorestoration and neuroprotection. This opens the door to the possibility that strategies directed at improving PLTs function could extend their benefits to the context of dementia [133] (Table 9).

In conclusion, the intricate relationship between platelet dysfunction, TBIs, and their consequences, underscores the need for comprehensive understanding and innovative interventions. The potential benefits of PLT transfusions and the promising avenues explored in HPL biotherapy encourage further research into improving outcomes for individuals grappling with traumatic brain injuries and the associated neurological complications (Table 10).

### 3.8. The Role of Platelets in Mild Cognitive Impairment

Mild Cognitive Impairment (MCI) serves as a transitional stage between normal aging and dementia [134], often recognised as the interval between typical cognitive function and the onset of AD [135]. Marked by the emergence of AD symptoms, MCI is considered an early stage of AD. Research indicates that individuals with MCI exhibit brain changes similar to those observed in AD [136]. Nevertheless, it is noteworthy that not all individuals diagnosed with MCI experience continuous cognitive decline or manifest additional symptoms of dementia [137].

MCI represents a condition characterised by relatively minor impairments in thought processes and memory, while AD is a distinct and progressive disorder where memory and functioning continually decline over time [135]. Given the anticipated increase in the incidence of AD in coming years, the early identification of MCI becomes crucial for preventing the progression of pathogenesis.

In recent years, attention has increasingly focused on PLTs concerning MCI, as they share many similarities with altered neurons in both MCI and AD. These cellular fragments may play a dual role in these pathologies by participating in the pathogenic process. Since alterations similar to those observed in neurons are evident in PLTs, these changes hold potential utility in the diagnosis and monitoring of therapies for MCI.

ADAM10 and BACE1, proteases that are intricately involved in the processing of APP and crucial for the formation of Aβ amyloid, have undergone extensive investigation with the objective of characterising MCI and identifying differences that could be useful in discriminating MCI from AD.

A study reported a decrease in ADAM10 levels in PLTs associated with MCI and AD, suggesting that this reduction could serve as a potential biomarker for these conditions [138]. Similar findings were observed in both MCI and AD cases. In a study, the investigation into whether the reduction in ADAM10 protein levels is associated with alterations in ADAM10 mRNA yielded negative results. This led to the hypothesis that different mechanisms might be involved in the reduction in ADAM10 protein levels [77]. Nevertheless, the consistent observation of reduced ADAM10 protein levels in both MCI and AD implies that this pathway may be altered in the early phases of the disease. Consequently, this parameter holds promise as an intriguing biomarker warranting further investigations.

The protease BACE1, involved in APP processing, has shown promising results in distinguishing between MCI and AD subjects [139]. However, further confirmation through a larger sample size is necessary to solidify this finding. 

In a study, the investigation into whether the reduction in ADAM10 protein levels is associated with alterations in ADAM10 mRNA yielded negative results. This led to the hypothesis that different mechanisms might be involved in the reduction in ADAM10 protein levels. At baseline, no significant differences were found between the MCI and control groups. However, cholesterol levels were lower in the MCI group compared to the control group. They calculated the ratio of total membrane-secretase activity to membrane cholesterol, discovering a significant elevation in the MCI group. Unfortunately, this result did not reveal significant differences between individuals with stable MCI and those who later progressed to AD [140].

In a study, the investigation into whether the reduction in ADAM10 protein levels is associated with alterations in ADAM10 mRNA yielded negative results. This led to the hypothesis that different mechanisms might be involved in the reduction in ADAM10 protein levels. Their findings revealed that soluble PLT lipids undergo alterations during the progression of AD. Additionally, they identified specific lyso-phosphatidylcholines that could serve as discriminative markers, enabling the differentiation between MCI and AD [86].

PLTs have been explored in connection with their involvement in the inflammatory pathway, revealing a higher trend of platelet-to-lymphocyte ratio (PLR) in both AD and MCI patients when compared to controls. However, no statistically significant differences between these pathologies were found [134]. In relation to the inflammatory process observed in MCI and the role of PLTs in this pathway, MPV and PDW exhibited significantly lower values in patients with AD compared to either MCI or controls [141]. It is important to note that the literature presents conflicting results regarding MPV in AD, emphasising the need for further investigations to clarify its utility in distinguishing MCI from AD.

The investigation into whether the reduction in ADAM10 protein levels is associated with alterations in ADAM10 mRNA yielded negative results. This led to the hypothesis that different mechanisms might be involved in the reduction in ADAM10 protein levels [142].

Phosphorylase activity 2 (PLA2), another extensively studied PLT parameter, plays a role in both the inflammatory process and membrane remodelling, as well as cellular signalling [143]. The levels of this enzyme in PLTs are viewed as reflective of the central nervous system, suggesting its potential as a therapeutic target in both MCI and AD. A study examined the modulatory effects of PLA2 in MCI under cognitive stimulation. It was shown that the total PLA2 correlates with the cognitive conditions of MCI, and cognitive stimulation acts selectively on subjects with dysregulated total PLA2 [143]. In a similar way, researchers explored whether decreased PLA2 activity at baseline, assuming PLA2 activity is reduced in AD and MCI, is associated with the progression of MCI to AD over a 4-year follow-up period. They found that intracellular PLA2 was decreased at baseline in both AD and MCI patients. MCI patients who progressed to AD during follow-up exhibited a lower activity of intracellular calcium-independent PLA2 at baseline [144]. Moreover, healthy subjects who progressed to MCI during follow-up displayed lower levels of secreted calcium-dependent PLA2 and cytosolic calcium-dependent PLA2 at baseline, suggesting the potential predictive value of these markers. 

Taken together, these findings support the notion that PLA2 values could serve as a therapeutic target and as a tool to assess therapeutic effects.

A study, the COCIMID protocol, was initiated to assess the effects of cilostazol, a drug commonly used for the secondary prevention of ischemic strokes, in preventing the progression of MCI to AD. The aim was to promote Aβ clearance and maintain cerebrovascular integrity to suppress cognitive decline. Despite patients tolerating the cilostazol well, the drug did not demonstrate significant effects in preventing cognitive decline [145].

In a related effort, a proteomic assay of platelets was conducted to identify potential biomarkers for MCI and AD. Four decreased proteins were identified, which have been recognised as promising indicators to predict cognitive decline [32]. However, further studies are needed to investigate whether these proteins could be valuable in the early screening of AD.

PLTs have also been investigated to identify potential risk factors indicative of the progression of type 2 diabetes mellitus (T2DM) in MCI. This study revealed a correlation between the increase in optineurin, an autophagy-related protein, and a reduction in the MMSE score. Additionally, the evaluation of optineurin levels proved effective in discriminating individuals with both T2DM and MCI from those with T2DM but not MCI [146] (Table 11 and Table 12).

### 3.9. Platelet Parameters as a Tool for Delirium Prediction

Delirium is characterised as an acute and fluctuating alteration in awareness and mentation, commonly observed in the context of serious medical illness [147]. The root cause of delirium is attributed to a decompensation of cerebral function in response to one or more pathophysiological stressors, such as infections, drugs, anoxia, and, in certain cases, prolonged anaesthesia [148]. Inflammation has been implicated as a potential etiological factor in the development of delirium in adult patients, particularly those undergoing surgery [147,149]. However, it is crucial to note that delirium is also a frequent, serious, and preventable complication in critically ill children [147].

The necessity for a predictive biomarker to aid in the prevention and identification of individuals predisposed to manifest delirium has prompted researchers to explore blood samples. This investigation has uncovered the potential utility of PLTs and their parameters. When used in conjunction with other blood and serum markers, these PLT parameters can contribute to the early identification of the possible onset of delirium.

Hypotheses suggesting that PLT transfusion might be a potential cause of delirium, both in adults and children, were not confirmed in subsequent studies [150]. However, noteworthy results have been achieved concerning PLT parameters.

Researchers identified the platelet-to-lymphocyte ratio (PLR) and platelet-to-white blood cell ratio (PWR), among other parameters, as statistically significant risk factors capable of predicting delirium onset in postoperative elderly patients following abdominal surgery [151]. Similar findings regarding PWR as a predictor of postoperative delirium have been reported by other research groups [152,153,154]. 

PLTs have been considered as inflammatory markers in delirium studies. Şaşkin et al. observed higher pre- and early postoperative MPV, PLR, and other inflammatory indicators in patients who developed postoperative delirium [149]. However, conflicting results exist regarding PLR. Jiang et al. reported a high PLR associated with a higher incidence of delirium upon admission to the intensive care unit [155], while another group did not find any significance in PLR levels between patients who manifested postoperative delirium and those who did not [156]. Soler-Sanchis et al. conducted a case-control study, discovering significantly lower values of PLTs, PLR, and MPV in patients compared to controls [157].

A study confirmed the role of PLR in the occurrence of postoperative delirium, while another study demonstrated the association of the severity of delirium with a decreased number of PLTs and MPV [148,158]. PLTs have also been investigated by analysing their mitochondrial mass after surgery, identifying a good predictive value for postoperative delirium [159].

Given the need for predictive and early markers of delirium, as it is a serious postoperative complication occurring in a significant percentage of patients [152], markers such as MPV, the neutrophil-to-lymphocyte ratio (NLR), PLR, and PWR, along with other blood parameters, appear useful in delirium prediction and can be easily obtained through routine laboratory analyses. However, the presence of conflicting results suggests the necessity to further explore and clarify this topic (Table 13 and Table 14).

### 3.10. Platelets in Huntington’s Disease

Huntington’s disease (HD) is a neurodegenerative disorder characterised by a mutation in the huntingtin gene, leading to the production of an abnormal protein known as mutant Huntington (mHtt). This mutation involves an expansion of CAG repeats in the exon 1 of the *HD* gene, resulting in an abnormal polyglutamine (polyQ) tract at the N-terminus of the Huntington protein.

The mutant Huntington protein tends to form insoluble aggregates and is responsible for the neuropathological signs of HD, particularly causing the selective death of striatal medium spiny neurons [18,160]. Clinically, HD is characterised by involuntary movements, psychiatric disturbances, and progressive dementia. Patients typically succumb to the disease within 15–20 years after its onset [160].

The mechanisms underlying neuronal death following the expansion of polyQ repeats still remain inconclusive, but mitochondrial dysfunction appears to play a pivotal role in the disease. Silva et al. investigated the activity of mitochondrial complexes (Cx) I–IV in PLTs isolated from pre-symptomatic and symptomatic HD carriers, as well as age-matched control individuals. Their findings revealed a reduced activity of citrate synthase in pre-symptomatic individuals and a reduced activity of Cx-I in both pre-symptomatic and symptomatic HD carriers [160].

The data obtained by Ehinger et al. align with those reported, indicating dysfunction in complex II and a lower maximum oxidative phosphorylation capacity in HD [161]. Despite limited research on PLTs in HD pathology, the study of peripheral systems in neurological disorders has gained attraction in recent years.

A study highlighted PLT dysfunction in HD, particularly in the release of angiogenic factors and functions related to thrombosis, angiogenesis, and vascular homeostasis. This suggests a potential involvement of platelets in the vascular abnormalities reported in HD brains [162].

A study was conducted on PLT activity, focusing on their role in the regulation of the vascular system and the modulation of nitric oxide (NO) signalling. They observed an impairment of endothelial nitric oxide synthase (eNOS) phosphorylation and activity, potentially contributing to defective vasorelaxation in HD. The decrease in NO in PLTs of HD individuals is proposed as a potential tool for monitoring advanced stages of the disease [163].

In an effort to identify pathological signatures in neurological disorders in peripheral blood, researchers analysed the extracellular vesicles derived from PLTs in HD. However, they concluded that, while these structures are interesting to study, they may not serve as useful pathological biomarkers [18] (Table 15 and Table 16).

## 4. Conclusions

In summary, this systematic review aimed to uncover the complex role of PLTs in neurocognitive disorders, highlighting their involvement in cognitive functions and the potential for the early identification of these disorders, which is crucial given the increasing prevalence of neurocognitive disorders among the elderly population and the significant economic burden associated with their management. The literature reviewed revealed that PLTs, traditionally known for their haemostatic roles, exhibit a broader range of functions, including physiological ones such as tissue remodelling, brain plasticity, and the secretion of neurotrophic factors. They also play a role in inflammation and the deposition of insoluble protein aggregates characteristic of pathologies like AD and HD.

The study of APP fractions and PLT parameters, such as MPV, has shown promise in distinguishing between diseased subjects and healthy controls. Moreover, these parameters have been found to correlate with the MMSE, suggesting their potential as surrogate biomarkers for assessing disease progression and treatment effectiveness. The evidence suggests that PLTs could serve as a reliable and non-invasive tool for monitoring neuropathological alterations in neurons.

However, it is important to acknowledge the presence of conflicting data in the literature, which may arise from variations in sample populations, methodologies, or disease stages. This highlights the need for further research and standardisation in assessing larger and more comprehensive studies, which are essential for ensuring the reliability and generalisability of findings in the context of these pathologies.

Despite the promising findings, the question of whether the observed modifications in PLTs are a consequence of neurological disorders or whether PLTs play a role in the pathogenesis of these diseases remains unanswered. This underscores the importance of continued investigation into the biological mechanisms underlying neurocognitive disorders and the potential of PLTs as diagnostic or prognostic biomarkers.

Future research should focus on developing valid, reliable, and broadly usable biomarkers that can support the timely diagnosis and management of neurocognitive disorders. This includes exploring models that include multiple biomarkers converging on the same or related biological pathways, as suggested by the findings across different areas of study. Additionally, future studies should aim to validate candidate biomarkers in external, independent samples and assess their feasibility and cost-effectiveness before implementation in clinical practice.

In conclusion, while the role of PLTs in neurocognitive disorders is complex and multifaceted, the evidence suggests that they could serve as valuable tools for early detection, monitoring, and management of these conditions. However, further research is needed to clarify the mechanisms underlying these relationships and to develop robust biomarkers for clinical use. 

## Figures and Tables

**Table 1:** Role of platelets as biomarkers in Alzheimer’s Disease (AD).

Reference	Disease	Samples	Study Objective	Results
Sun et al. (2018) [19]	AD	58 AD 61 HCs	Evaluation of the correlation between the levels of blood-derived amyloid-beta (Aβ) and the count of platelets.	The plasma Aβ42 level was positively correlated with the platelet count in both AD patients and control group.
Fisar et al. (2019) [60]	AD	50 AD 15 vascular dementia 25 HCs	Analysis of the associations between disturbed platelet mitochondrial respiration and plasma concentrations of Aβ40 and Aβ42 in patients with AD.	Aβ40, Aβ42, and Aβ42/Aβ40 levels were not significantly altered in patients with AD compared with controls; the association between mitochondrial respiration in platelets and plasma Aβ levels differs in patients with AD and controls.
Jelic et al. (2013) [57]	AD	30 AD 23 elderly subjects	Exploration of the diagnostic potential of platelet AβPP metabolism.	AβPP 115 kDa species exhibited an increase in PLTs in AD compared to control subjects. There is a correlation between the abundance of the AβPP 115 kDa species and Mini-Mental State Examination scores.
Vignini et al. (2013) [68]	Frontotemporal lobar degeneration (FTLD) AD	20 AD 19 FTLD 18 HCs	Analysis of the mRNA expression level of Total APP (TOT) and APP containing a Kunitz-type serine protease inhibitor domain (KPI) in the platelets of AD patients, FTLD, and HCs. Evaluation of the correlation between platelet APP mRNA expression levels and cognitive impairment.	Significant upregulation of both APP TOT and APP KPI in the PLTs of patients with both AD and FTLD compared to HCs. In FTLD patients, this expression did not correlate with the severity of cognitive impairment.
Srisawat et al. (2013) [69]	AD	13 AD 27 HCs	Evaluation of the role of the platelet amyloid precursor protein (APP) ratio as diagnostic marker for (AD).	No significance difference between AD and HCs subjects.
Wilhite et al. (2017) [73]	AD	10 AD 10 HCs	Evaluation of the TDP-43 profile in PLTs in order to use TDP_43 as a surrogate biomarker for AD screening.	Increased TDP-43 in post mortem AD brain regions and also in patient’s platelets.
Bram et al. (2019) [74]	AD	20 AD 20 HCs	Comparison of the protein level of the APP secretases A-disintegrin, ADAM10, BACE1, PSEN1 in platelets and leukocytes of AD and HCs.	Significant decrease in ADAM10 and PSEN1 in platelets from AD patients compared to controls. The combination of protein levels of ADAM10, BACE1, and PSEN1 in platelets, demonstrated good accuracy in discriminating AD from controls.
Sarg et al. (2022) [66]	AD Mild cognitive impairment (MCI)	15 AD 15 MCI 15 HCs	Characterisation of the molecular appearance of tau and examination of its alterations in patients with neurocognitive impairment.	Reduction in tau protein in AD patients compared to healthy subjects.
Slachevsky et al. (2017) [20]	AD	53 AD 37 HCs	Determining whether (HMW) or (LMW) tau protein levels, as well as the ratio HMW/LMW present in platelets correlates with brain magnetic resonance imaging (MRI) structural changes in HCs and AD subjects.	HMW/LMW tau ratio was statistically different between controls and AD patients and it is associated with specific brain regions atrophy.
Koc et al. (2014) [83]	AD	109 AD 81 HCs	Examination of the role of MPV as a marker of vascular damage in AD.	MPV levels were higher in the AD group.

**Table 2:** GRADE criteria for the risk-of-bias evaluation for the role of platelets as biomarkers in Alzheimer’s disease (AD). Green: no risk of bias; yellow: low or potential risk of bias.

Authors (Year)	Reference	Methodological Quality	Directness of Evidence	Heterogeneity	Precision of Effect Estimates	Publication Bias	Overall Quality of Evidence
Sun et al. (2018)	[19]						
Fisar et al. (2019)	[60]						
Jelic et al. (2013)	[57]						
Vignini et al. (2013)	[68]						
Srisawat et al. (2013)	[69]						
Wilhite et al. (2017)	[73]						
Bram et al. (2019)	[74]						
Sarg et al. (2022)	[66]						
Slachevsky et al. (2017)	[20]						
Koc et al. (2014)	[83]						

**Table 3:** Role of platelet in Parkinson’s disease (PD).

Reference	Disease	Samples/Animal Models	Study Objective	Results
Zhang L. et al. (2022) [104]	PD	Blood samples of PD patients and HCs	Identification of 2221 differential transcript levels between PD and HCs through RNA sequencing on platelet.	Whilst 1041 genes are upregulated, 1180 are downregulated. WASH5P is the most upregulated gene, and AC114491.1 is the most downregulated. MALAT1, EEF1A1, and CTSS were associated with PD.
Montenegro et al. (2022) [105]	PD	Platelets 70 patients 113 HCs	Secretory vesicles (SVs) are fundamental in the regulation of cytosolic DA levels. SVs of platelets use similar. Functional defects in platelets probably mirror events in DA neurons.	Dramatic decrease in the serotonin content and uptake by SVs, decreased thrombin-induced release by platelets from PD patients. Platelets from PD patients also failed to retain serotonin in SVs. This was not observed in most cases of Parkinsonism.
Page et al. (2022) [106]	AD PD		Role of platelet in the mechanism of neurodegeneration.	Platelets are implicated in the pathological processes of inflammation, neurovascular dysfunction, and coagulopathy. These processes contribute to the progression of neurodegenerative diseases
Özdemir et al. (2021) [107]	AD PD		Effect of MAO-B inhibitors in the treatment of PD and AD.	MAO-B activity increases with age in brain tissue, cerebrospinal fluid (CSF), and platelets in Alzheimer’s patients.
Jiang L. et al. (2022) [108]	PD	47 MSA 125 PD 124 HCs	Exploring the association between inflammatory markers (MHR, NLR, and RPR) and MSA, and the difference between MSA and Parkinson’s disease.	The red-cell-distribution-width-to-platelet ratio (RDW-PR) was found to be higher in both the MSA group and the PD group compared to the HCs group. This suggests that inflammation, often present in MSA and PD, could potentially affect platelet numbers.
Beura et al. (2023) [109]	PD		Investigating the impact of 6-hydroxydopamine (6-OHDA) on platelet function.	6-OHDA treatment increased the production of ROS in human blood platelets. This ROS production seems to be regulated by the IP3 receptor-Ca^2+^-NOX signalling axis in human blood platelets.
Bronstein et al. (2015) [110]	PD	23 PD 23 HCs	Examination of the role of platelet mitochondrial activity and exposure to pesticides in the early stages of PD.	The study found no differences in complex I and I/III activities in subjects with PD and controls. It did find that NCCR activity correlated with subjects’ exposure to pesticides known to inhibit mitochondrial activity regardless of their diagnosis.
Delila et al. (2022) [111]	PD		Study of the potential neuroprotective effects on dopaminergic neurons of platelet lysate.	The platelet lysate maintained its neuroprotective activity in a Lund human mesencephalic dopaminergic neuron model of PD disease.
Tirozzi et al. (2023) [112]	Major depressive disorder (MDD), AD, PD		Analysis of genetic factors associated with brain disorders like Alzheimer’s disease (AD), Parkinson’s disease (PD), and Major Depressive Disorder (MDD) in relation to platelet traits.	The most significant associations were detected in some of the most implicated genes in neurodegenerative/neuropsychiatric disorders, APOE with AD, SNCA with PD and ZSCAN12 with MDD.
Stanca et al. (2022) [113]	PD	45 PD 46 HCs	Evaluation of the role of ESR, NLR, and PLR as prognostic factors in PD patients.	ESR did not show statistically significant correlations with motor score or with disability; ESR was correlated with the disease duration and PLR showed a significant correlation with disease stage and disease duration.

**Table 4:** GRADE criteria for risk-of-bias evaluation for role of platelet in Parkinson’s disease (PD). Green: no risk of bias; yellow: low or potential risk of bias.

Authors (Year)	Reference	Methodological Quality	Directness of Evidence	Heterogeneity	Precision of Effect Estimates	Publication Bias	Overall Quality of Evidence
Zhang L. et al. (2022)	[104]						
Montenegro et al. (2022)	[105]						
Page et al. (2022)	[106]						
Özdemir et al. (2021)	[107]						
Jiang L. et al. (2022)	[108]						
Beura et al. (2023)	[109]						
Bronstein et al. (2015)	[110]						
Delila et al. (2022)	[111]						
Tirozzi et al. (2023)	[112]						
Stanca et al. (2022)	[113]						

**Table 6:** GRADE criteria for risk-of-bias evaluation for the role of platelet as biomarkers in vascular dementia (VD). Green: no risk of bias; yellow: low or potential risk of bias.

Authors (Year)	Reference	Methodological Quality	Directness of Evidence	Heterogeneity	Precision of Effect Estimates	Publication Bias	Overall Quality of Evidence
Albrecht et al. (2020)	[115]						
Qing-Cheng Liang et al. (2014)	[116]						
Palix et al. (2022)	[84]						
Bayat et al. (2020) [123]	[123]						
Schröder et al. (2022) [118]	[118]						
Donner et al. (2016)	[119]						
Pan et al. (2022)	[124]						
Tayler et al. (2023)	[120]						
Socha et al. (2019)	[114]						
Narasimhalu et al. (2015) [122]	[122]						

**Table 7:** Role of platelet as biomarkers in frontotemporal lobar degeneration (FTLD).

Reference	Disease	Samples	Study Objective	Results
Schröder S et al. (2022) [118]	Different dementia types	97 patients	Potential role of WBC and platelet counts as biomarkers in dementia diagnostics.	No significant differences across diverse dementia types.
Fourier et al. (2019) [125]	Frontotemporal lobar degeneration syndrome	9 patients	Developing an automated Simple Western assay to detect and quantify TDP43 protein.	Simple Western assay seems to be suitable for detecting and quantifying TDP43 protein in platelet samples, providing a potential candidate biomarker in this disease.

**Table 8:** GRADE criteria for risk-of-bias evaluation for platelet as biomarkers in frontotemporal lobar degeneration (FTLD). Green: no risk of bias; yellow: low or potential risk of bias.

Authors (Year)	Reference	Methodological Quality	Directness of Evidence	Heterogeneity	Precision of Effect Estimates	Publication Bias	Overall Quality of Evidence
Schröder S et al. (2022)	[118]						
Fourier et al. (2019)	[125]						

**Table 9:** Role of platelets in traumatic brain injury (TBI).

Reference	Disease	Samples	Study Objective	Results
Jehan et al. (2019) [128]	TBI	243 TBI	Evaluation of outcomes after TBI in patients received a PLT transfusion.	PLT transfusion was associated with decreased risk of progression of intracranial haemorrhage, neurosurgical intervention, and mortality.
Davis et al. (2013) [129]	TBI	50 TBI 50 HCs	Evaluation of association between PLT disfunction and TBI.	Significantly increased percentage of platelet ADP and arachidonic acid (AA) receptor inhibition in patients with TBI. ADP inhibition correlates strongly with severity of TBI and mortality.
Nekludov et al. (2007) [131]	TBI	20 TBI 10 HCs	Investigation of PLT function in patients with TBI.	TBI patients had a lower PLT count, a long bleeding time, and lower PLT responses to AA, correlated with an elevated risk of bleeding complications.

**Table 10:** GRADE criteria for risk-of-bias evaluation for role of platelets in traumatic brain injury (TBI). Green: no risk of bias; yellow: low or potential risk of bias; red: high risk of bias.

Authors (Year)	Reference	Methodological Quality	Directness of Evidence	Heterogeneity	Precision of Effect Estimates	Publication Bias	Overall Quality of Evidence
Jehan et al. (2019)	[128]						
Davis et al. (2013)	[129]						
Nekludov et al. (2007)	[131]						

**Table 11:** Role of platelets as biomarkers in mild cognitive impairment (MCI).

Reference	Disease	Samples	Study Objective	Results
Vatanabe et al. (2013) [138]	MCI	61 MCI 61 HCs	Role of the platelet and plasma levels of ADAM10 as a biomarker in MCI and physical frailty.	Decrease in ADAM10 levels in platelets associated with mild cognitive impairment (MCI) and Alzheimer’s disease (AD).
Bermejo-Bescós et al. (2013) [139]	MCI AD	34 MCI 45 AD 28 HCs	Assessment of the status of the platelet amyloid precursor protein (APP) metabolism in MCI and AD.	Reduction in APP levels in MCI and AD vs. HCs; high levels of ADAM-10 in both MCI and AD vs. HCs. Higher ratio ADAM-10/BACE1 for the MCI group vs. AD group. BACE1 and PS1 levels were increased in AD vs. HCs BACE1 and γ-secretase activities significantly augmented in both MCI and AD groups.
Oberacher et al. (2017) [86]	MCI AD	37 AD 21 MCI 32 HCs	Study of the PLT proteome in patients with AD and MCI.	Soluble platelet lipids undergo alterations during the progression of AD. Identified platelet PCaeC40: as a potential marker to diagnose AD.
Wang et al. (2013) [141]	MCI AD	120 MCI 120 AD 120 HCs	Investigation of the relationship between platelet indices and MCI and AD pathogenesis.	MPV and PDW were significantly lower in patients with AD as compared with MCI or controls.
Zhang et al. (2023) [142]	MCI	22 MCI	Investigation of the role of complete blood counts in the relationship between brain function and cognitive performance.	PLT count, appears to be inversely correlated with cognitive functions.
Balietti et al. (2016) [143]	MCI	70 MCI 74 HCs	Evaluation of the effect of cognitive stimulation on platelet total phospholipases A2 activity (tPLA2A).	Total PLA2 correlates with the cognitive conditions of MCI, and cognitive stimulation acts selectively on subjects with dysregulated total PLA2.
Gattaz et al. (2014) [144]	MCI	44 AD 59 MCI 66 HCs	Investigating whether decreased PLA₂ activity at baseline is associated with the progression of MCI to AD upon a follow-up of 4 years.	MCI patients who progressed to AD exhibited a lower activity of intracellular calcium-independent PLA2 at baseline.

**Table 12:** GRADE criteria for risk-of-bias evaluation for the role of platelets as biomarkers in mild cognitive impairment (MCI). Green: no risk of bias; yellow: low or potential risk of bias.

Authors (Year)	Reference	Methodological Quality	Directness of Evidence	Heterogeneity	Precision of Effect Estimates	Publication Bias	Overall Quality of Evidence
Vatanabe et al. (2013)	[138]						
Bermejo-Bescós et al. (2013)	[139]						
Oberacher et al. (2017)	[86]						
Wang et al. (2013)	[141]						
Zhang et al. (2023)	[142]						
Balietti et al. (2016)	[143]						
Gattaz et al. (2014)	[144]						

**Table 13:** Role of platelets as biomarkers in delirium.

Reference	Disease	Samples	Study Objective	Results
Ida et al. (2020) [151]	Delirium	833	Identifying delirium risk factors and developing a predictive model using preoperative and intraoperative data.	(PLR) and platelet-to-white blood cell ratio (PWR) are associated with the development of postoperative delirium.
Şaşkin et al. (2022) [149]	Delirium	1279	Evaluating the association of pre-operative and early postoperative inflammatory parameters with postoperative delirium.	Higher pre- and early postoperative mean platelet volume, platelet-to-lymphocyte ratio.
Jiang et al. (2020) [155]	Delirium	319	Investigation of the predictive value of the platelet-to-lymphocyte ratio for delirium in the intensive care unit.	High PLR associated with a higher incidence of delirium upon admission to the intensive care unit.
Soler-Sachis et al. (2022) [157]	Delirium	128 delirium 128 HCS	Identification of biomarkers included in standard blood examinations in patients with delirium in the emergency department.	Lower values of PLTs, PLR, and MPV in patients compared to controls.
Thisayacorn et al. (2021) [158]	Delirium	65	Exploration of the associations between delirium and its features and immune-inflammatory and blood gas biomarkers.	Association of the severity of delirium with a decreased number of platelets and MPV.

**Table 14:** GRADE criteria for risk-of-bias evaluation for the role of platelets as biomarkers in delirium. Green: no risk of bias; yellow: low or potential risk of bias.

Authors (Year)	Reference	Methodological Quality	Directness of Evidence	Heterogeneity	Precision of Effect Estimates	Publication Bias	Overall Quality of Evidence
Mitsuru et al. (2020)	[151]						
Şaşkin et al. (2022)	[149]						
Jiang et al. (2020)	[155]						
Soler-Sachis et al. (2022)	[157]						
Thisayacorn et al. (2021)	[158]						
Şaşkin et al. (2022)	[149]						

**Table 15:** Role of platelets as biomarkers in Huntington’s disease (HD).

Reference	Disease	Samples	Study Objective	Results
Silva et al. (2013) [160]	HD	7 HD symptomatic patients 8 pre-HD carriers 8 HCs	Investigating the activity of mitochondrial complexes (Cx) I–IV in platelets isolated from pre-symptomatic and symptomatic HD carriers.	Reduced activity of citrate synthase in pre-symptomatic individuals and reduced activity of Cx-I in both pre-symptomatic and symptomatic HD carriers.
Ehinger et al. (2016) [161]	HD	14 HD 21 HCs	Investigating the role of mitochondrial complex I, complex II function, and maximum oxidative phosphorylation capacity in HD.	Platelets from patients with HD displayed respiratory dysfunction linked to complex I, complex II, and lower maximum oxidative phosphorylation capacity.
Denis et al. (2019) [162]	HD	71 HD 71 HCs	Investigating the involvement of PLTs in the vascular abnormalities reported in HD brains.	Platelets in HD patients are dysfunctional.
Carrizzo et al. (2014) [163]	HD	55 HD 28 HCs	Investigating PLT role in the regulation of the vascular system and modulation of nitric oxide (NO) signalling.	Decrease in NO in platelets of HD individuals could be correlated with advanced stages of the disease.

**Table 16:** GRADE criteria for risk-of-bias evaluation for the role of platelets as biomarkers in Huntington’s disease (HD). Green: no risk of bias; yellow: low or potential risk of bias.

Authors (Year)	Reference	Methodological Quality	Directness of Evidence	Heterogeneity	Precision of Effect Estimates	Publication Bias	Overall Quality of Evidence
Silva et al. (2013)	[160]						
Ehinger et al. (2016)	[161]						
Denis et al. (2019)	[162]						
Carrizzo et al. (2014)	[163]						

## Data Availability

Not applicable.

## References

[1] McDonald W.M. (2017). Overview of neurocognitive disorders. Focus.

[2] Strydom A., Fleisher M.H., Deb S., Ring H., Esralew L., Dodd K., al Janab T., Trollor J., Whitwham S.L. (2016). Neurocognitive Disorders.

[3] Wojsiat J., Laskowska-Kaszub K., Mietelska-Porowska A., Wojda U. (2017). Search for alzheimer’s disease biomarkers in blood cells: Hypotheses-driven approach. Biomark. Med..

[4] Hou Y., Dan X., Babbar M., Wei Y., Hasselbalch S.G., Croteau D.L., Bohr V.A. (2019). Ageing as a risk factor for neurodegenerative disease. Nat. Rev. Neurol..

[5] Kim S., Kim D.K., Jeong S., Lee J. (2022). The common cellular events in the neurodegenerative diseases and the associated role of endoplasmic reticulum stress. Int. J. Mol. Sci..

[6] Stephenson J., Nutma E., van der Valk P., Amor S. (2018). Inflammation in cns neurodegenerative diseases. Immunology.

[7] Wilson D.M., Cookson M.R., Van Den Bosch L., Zetterberg H., Holtzman D.M., Dewachter I. (2023). Hallmarks of neurodegenerative diseases. Cell.

[8] Mayne K., White J.A., McMurran C.E., Rivera F.J., de la Fuente A.G. (2020). Aging and neurodegenerative disease: Is the adaptive immune system a friend or foe?. Front. Aging Neurosci..

[9] Hammond T.R., Marsh S.E., Stevens B. (2019). Immune signaling in neurodegeneration. Immunity.

[10] Dominguez L.J., Veronese N., Vernuccio L., Catanese G., Inzerillo F., Salemi G., Barbagallo M. (2021). Nutrition, physical activity, and other lifestyle factors in the prevention of cognitive decline and dementia. Nutrients.

[11] Lopreite M., Mauro M. (2017). The effects of population ageing on health care expenditure: A bayesian var analysis using data from italy. Health Policy.

[12] Deb A., Thornton J.D., Sambamoorthi U., Innes K. (2017). Direct and indirect cost of managing alzheimer’s disease and related dementias in the united states. Expert. Rev. Pharmacoeconomics Outcomes Res..

[13] Dieleman J.L., Cao J., Chapin A., Chen C., Li Z., Liu A., Horst C., Kaldjian A., Matyasz T., Scott K.W. (2020). Us health care spending by payer and health condition, 1996–2016. JAMA.

[14] Wareham L.K., Liddelow S.A., Temple S., Benowitz L.I., Di Polo A., Wellington C., Goldberg J.L., He Z., Duan X., Bu G. (2022). Solving neurodegeneration: Common mechanisms and strategies for new treatments. Mol. Neurodegener..

[15] Jones K.C. (2021). Update on major neurocognitive disorders. Focus.

[16] Coradduzza D., Sedda S., Cruciani S., De Miglio M.R., Ventura C., Nivoli A., Maioli M. (2023). Age-related cognitive decline, focus on microbiome: A systematic review and meta-analysis. Int. J. Mol. Sci..

[17] Zhang W., Xiao D., Mao Q., Xia H. (2023). Role of neuroinflammation in neurodegeneration development. Signal Transduct. Target. Ther..

[18] Denis H.L., Lamontagne-Proulx J., St-Amour I., Mason S.L., Weiss A., Chouinard S., Barker R.A., Boilard E., Cicchetti F. (2018). Platelet-derived extracellular vesicles in huntington’s disease. J. Neurol..

[19] Sun H.-L., Li W.-W., Zhu C., Jin W.-S., Liu Y.-H., Zeng F., Wang Y.-J., Bu X.-L. (2018). The correlations of plasma and cerebrospinal fluid amyloid-beta levels with platelet count in patients with alzheimer’s disease. BioMed. Res. Int..

[20] Slachevsky A., Guzmán-Martínez L., Delgado C., Reyes P., Farías G.A., Muñoz-Neira C., Bravo E., Farías M., Flores P., Garrido C. (2017). Tau platelets correlate with regional brain atrophy in patients with alzheimer’s disease. J. Alzheimer’s Dis..

[21] Cadoni M.P.L., Biggio M.L., Arru G., Secchi G., Orrù N., Clemente M.G., Sechi G., Yamoah A., Tripathi P., Orrù S. (2020). Vapb er-aggregates, a possible new biomarker in als pathology. Cells.

[22] Gorji A. (2022). Neuroinflammation: The pathogenic mechanism of neurological dfisorders. Int. J. Mol. Sci..

[23] Owens T., Bechmann I., Engelhardt B. (2008). Perivascular spaces and the two steps to neuroinflammation. J. Neuropathol. Exp. Neurol..

[24] Lyman M., Lloyd D.G., Ji X., Vizcaychipi M.P., Ma D. (2014). Neuroinflammation: The role and consequences. Neurosci. Res..

[25] Jorfi M., Maaser-Hecker A., Tanzi R.E. (2023). The neuroimmune axis of alzheimer’s disease. Genome Med..

[26] Arru G., Galleri G., Deiana G.A., Zarbo I.R., Sechi E., Bo M., Cadoni M.P.L., Corda D.G., Frau C., Simula E.R. (2021). Herv-k modulates the immune response in als patients. Microorganisms.

[27] Arabi T.Z., Alabdulqader A.A., Sabbah B.N., Ouban A. (2023). Brain-inhabiting bacteria and neurodegenerative diseases: The brain microbiome theory. Front. Aging Neurosci..

[28] Fend F., van den Brand M., Groenen P.J., Quintanilla-Martinez L., Bagg A. (2023). Diagnostic and prognostic molecular pathology of lymphoid malignancies. Virchows Arch..

[29] Coradduzza D., Bo M., Congiargiu A., Azara E., De Miglio M.R., Erre G.L., Carru C. (2023). Decoding the microbiome’s influence on rheumatoid arthritis. Microorganisms.

[30] He T., Kaplan S., Kamboj M., Tang Y.-W. (2016). Laboratory diagnosis of central nervous system infection. Curr. Infect. Dis. Rep..

[31] Mietto B.S., Mostacada K., Martinez A.M.B. (2015). Neurotrauma and inflammation: Cns and pns responses. Mediat. Inflamm..

[32] Yu H., Liu Y., He B., He T., Chen C., He J., Yang X., Wang J.Z. (2021). Platelet biomarkers for a descending cognitive function: A proteomic approach. Aging Cell.

[33] Thon J.N., Italiano J.E. (2012). Platelets: Production, morphology and ultrastructure. Antiplatelet Agents.

[34] Leiter O., Walker T.L. (2019). Platelets: The missing link between the blood and brain?. Prog. Neurobiol..

[35] Coradduzza D., Garroni G., Congiargiu A., Balzano F., Cruciani S., Sedda S., Nivoli A., Maioli M. (2022). Micrornas, stem cells in bipolar disorder, and lithium therapeutic approach. Int. J. Mol. Sci..

[36] Coradduzza D., Cruciani S., Arru C., Garroni G., Pashchenko A., Jedea M., Zappavigna S., Caraglia M., Amler E., Carru C. (2022). Role of mirna-145, 148, and 185 and stem cells in prostate cancer. Int. J. Mol. Sci..

[37] Periayah M.H., Halim A.S., Saad A.Z.M. (2017). Mechanism action of platelets and crucial blood coagulation pathways in hemostasis. Int. J. Hematol.-Oncol. Stem Cell Res..

[38] Wen X., Coradduzza D., Shen J., Scanu A.M., Muroni M.R., Massidda M., Rallo V., Carru C., Angius A., De Miglio M.R. (2023). Harnessing minimal residual disease as a predictor for colorectal cancer: Promising horizons amidst challenges. Medicina.

[39] Golebiewska E.M., Poole A.W. (2015). Platelet secretion: From haemostasis to wound healing and beyond. Blood Rev..

[40] Leiter O., Walker T.L. (2020). Platelets in neurodegenerative conditions—Friend or foe?. Front. Immunol..

[41] Brożek J., Akl E.A., Alonso-Coello P., Lang D., Jaeschke R., Williams J.W., Phillips B., Lelgemann M., Lethaby A., Bousquet J. (2009). Grading quality of evidence and strength of recommendations in clinical practice guidelines: Part 1 of 3. An overview of the grade approach and grading quality of evidence about interventions. Allergy.

[42] Coradduzza D., Congiargiu A., Chen Z., Cruciani S., Zinellu A., Carru C., Medici S. (2023). Humanin and its pathophysiological roles in aging: A systematic review. Biology.

[43] Brignardello-Petersen R., Tomlinson G., Florez I., Rind D.M., Chu D., Morgan R., Mustafa R.A., Schünemann H., Guyatt G.H., GRADE Working Group (2023). Grading of recommendations assessment, development, and evaluation concept article 5: Addressing intransitivity in a network meta-analysis. J. Clin. Epidemiol..

[44] Coradduzza D., Medici S., Chessa C., Zinellu A., Madonia M., Angius A., Carru C., De Miglio M.R. (2023). Assessing the predictive power of the hemoglobin/red cell distribution width ratio in cancer: A systematic review and future directions. Medicina.

[45] Chen Z., Wang J., Carru C., Coradduzza D., Li Z. (2023). The prevalence of depression among parents of children/adolescents with type 1 diabetes: A systematic review and meta-analysis. Front. Endocrinol..

[46] Page M.J., McKenzie J.E., Bossuyt P.M., Boutron I., Hoffmann T.C., Mulrow C.D., Shamseer L., Tetzlaff J.M., Akl E.A., Brennan S.E. (2021). The prisma 2020 statement: An updated guideline for reporting systematic reviews. Int. J. Surg..

[47] Stojkovska I., Wani W.Y., Zunke F., Belur N.R., Pavlenko E.A., Mwenda N., Sharma K., Francelle L., Mazzulli J.R. (2022). Rescue of α-synuclein aggregation in parkinson’s patient neurons by synergistic enhancement of er proteostasis and protein trafficking. Neuron.

[48] Zhang Z., Yang X., Song Y.-Q., Tu J. (2021). Autophagy in alzheimer’s disease pathogenesis: Therapeutic potential and future perspectives. Ageing Res. Rev..

[49] Ross C.A., Poirier M.A. (2004). Protein aggregation and neurodegenerative disease. Nat. Med..

[50] Arrasate M., Finkbeiner S. (2012). Protein aggregates in huntington’s disease. Exp. Neurol..

[51] Stollings J.L., Kotfis K., Chanques G., Pun B.T., Pandharipande P.P., Ely E.W. (2021). Delirium in critical illness: Clinical manifestations, outcomes, and management. Intensive Care Med..

[52] Coradduzza D., Congiargiu A., Chen Z., Zinellu A., Carru C., Medici S. (2023). Ferroptosis and senescence: A systematic review. Int. J. Mol. Sci..

[53] Rawish E., Nording H., Münte T., Langer H.F. (2020). Platelets as mediators of neuroinflammation and thrombosis. Front. Immunol..

[54] Pluta R., Ułamek-Kozioł M., Januszewski S., Czuczwar S. (2018). Platelets, lymphocytes and erythrocytes from alzheimer’s disease patients: The quest for blood cell-based biomarkers. Folia Neuropathol..

[55] Wang Q., Shi Y., Qi X., Qi L., Chen X., Shi J., Xie C., Zhang Z. (2022). Platelet-derived amyloid-β protein precursor as a biomarker of alzheimer’s disease. J. Alzheimer’s Dis..

[56] Marksteiner J., Humpel C. (2013). Platelet-derived secreted amyloid-precursor protein-β as a marker for diagnosing alzheimer’s disease. Curr. Neurovascular Res..

[57] Jelic V., Hagman G., Yamamoto N.G., Teranishi Y., Nishimura T., Winblad B., Pavlov P.F. (2013). Abnormal platelet amyloid-β protein precursor (aβpp) metabolism in alzheimer’s disease: Identification and characterization of a new aβpp isoform as potential biomarker. J. Alzheimer’s Dis..

[58] Liu L., Zhang K., Tan L., Chen Y.-H., Cao Y.-P. (2015). Alterations in cholesterol and ganglioside gm1 content of lipid rafts in platelets from patients with alzheimer disease. Alzheimer Dis. Assoc. Disord..

[59] Bacchetti T., Vignini A., Giulietti A., Nanetti L., Provinciali L., Luzzi S., Mazzanti L., Ferretti G. (2015). Higher levels of oxidized low density lipoproteins in alzheimer’s disease patients: Roles for platelet activating factor acetyl hydrolase and paraoxonase-1. J. Alzheimer’s Dis..

[60] Fisar Z., Hroudová J., Hansíková H., Lelková P., Wenchich L., Jirák R., Zeman J., Martásek P., Raboch J. (2016). Mitochondrial respiration in the platelets of patients with alzheimer’s disease. Curr. Alzheimer Res..

[61] Wang X., Liu G.J., Gao Q., Li N., Wang R.T. (2020). C-type lectin-like receptor 2 and zonulin are associated with mild cognitive impairment and alzheimer’s disease. Acta Neurol. Scand..

[62] Thal D.R., Griffin W.S.T., de Vos R.A., Ghebremedhin E. (2008). Cerebral amyloid angiopathy and its relationship to alzheimer’s disease. Acta Neuropathol..

[63] Gowert N.S., Donner L., Chatterjee M., Eisele Y.S., Towhid S.T., Münzer P., Walker B., Ogorek I., Borst O., Grandoch M. (2014). Blood platelets in the progression of alzheimer’s disease. PLoS ONE.

[64] Bloom G.S. (2014). Amyloid-β and tau: The trigger and bullet in alzheimer disease pathogenesis. JAMA Neurol..

[65] Wang Z., Zheng Y., Cai H., Yang C., Li S., Lv H., Feng T., Yu Z. (2023). Aβ1-42-containing platelet-derived extracellular vesicle is associated with cognitive decline in parkinson’s disease. Front. Aging Neurosci..

[66] Sarg B., Korde D.S., Marksteiner J., Humpel C. (2022). Platelet tau is associated with changes in depression and alzheimer’s disease. Front. Biosci. -Landmark.

[67] Fišar Z., Jirák R., Zvěřová M., Setnička V., Habartová L., Hroudová J., Vaníčková Z., Raboch J. (2019). Plasma amyloid beta levels and platelet mitochondrial respiration in patients with alzheimer’s disease. Clin. Biochem..

[68] Vignini A., Morganti S., Salvolini E., Sartini D., Luzzi S., Fiorini R., Provinciali L., Di Primio R., Mazzanti L., Emanuelli M. (2013). Amyloid precursor protein expression is enhanced in human platelets from subjects with alzheimer’s disease and frontotemporal lobar degeneration: A real-time pcr study. Exp. Gerontol..

[69] Srisawat C., Junnu S., Peerapittayamongkol C., Futrakul A., Soi-ampornkul R., Senanarong V., Praditsuwan R., Assantachai P., Neungton N. (2013). The platelet amyloid precursor protein ratio as a diagnostic marker for alzheimer’s disease in thai patients. J. Clin. Neurosci..

[70] Srisawat N., WKulvichit, Mahamitra N., Hurst C., Praditpornsilpa K., Lumlertgul N., Chuasuwan A., Trongtrakul K., Tasnarong A., Champunot R. (2020). The epidemiology and characteristics of acute kidney injury in the southeast asia intensive care unit: A prospective multicentre study. Nephrol. Dial. Transplant..

[71] Casoli T., Giuli C., Balietti M., Fabbietti P., Conti F. (2020). Effect of a cognitive training program on the platelet app ratio in patients with alzheimer’s disease. Int. J. Mol. Sci..

[72] Chatterjee P., Gupta V.B., Fagan A.M., Jasielec M.S., Xiong C., Sohrabi H.R., Dhaliwal S., Taddei K., Bourgeat P., Brown B.M. (2015). Decreased platelet app isoform ratios in autosomal dominant alzheimer’s disease: Baseline data from a dian cohort subset. Curr. Alzheimer Res..

[73] Wilhite R., Sage J.M., Bouzid A., Primavera T., Agbas A. (2017). Platelet phosphorylated tdp-43: An exploratory study for a peripheral surrogate biomarker development for alzheimer’s disease. Future Sci. OA.

[74] Bram J.M.d.F., Talib L.L., Joaquim H.P.G., Sarno T.A., Gattaz W.F., Forlenza O.V. (2019). Protein levels of adam10, bace1, and psen1 in platelets and leukocytes of alzheimer’s disease patients. Eur. Arch. Psychiatry Clin. Neurosci..

[75] Angius A., Pira G., Cossu-Rocca P., Sotgiu G., Saderi L., Muroni M.R., Virdis P., Piras D., Vincenzo R., Carru C. (2023). Deciphering clinical significance of bcl11a isoforms and protein expression roles in triple-negative breast cancer subtype. J. Cancer Res. Clin. Oncol..

[76] Decourt B., Walker A., Gonzales A., Malek-Ahmadi M., Liesback C., Davis K.J., Belden C.M., Jacobson S.A., Sabbagh M.N. (2013). Can platelet bace1 levels be used as a biomarker for alzheimer’s disease? Proof-of-concept study. Platelets.

[77] Manzine P.R., Marcello E., Borroni B., Kamphuis W., Hol E., Padovani A., Nascimento C.C., de Godoy Bueno P., Vale F.A.C., Pavarini S.C.I. (2015). Adam10 gene expression in the blood cells of alzheimer’s disease patients and mild cognitive impairment subjects. Biomarkers.

[78] Manzine P.R., Barham E.J., Vale F.A.C., Selistre-de-Araújo H.S., Pavarini S.C.I., Cominetti M.R. (2014). Platelet a disintegrin and metallopeptidase 10 expression correlates with clock drawing test scores in alzheimer’s disease. Int. J. Geriatr. Psychiatry.

[79] Manzine P.R., Barham E.J., Vale F.D.A.C.D., Selistre-de-Araujo H.S., Pavarini S.C.I., Cominetti M.R. (2013). Correlation between mini-mental state examination and platelet adam10 expression in alzheimer’s disease. J. Alzheimer’s Dis..

[80] Strijkova V., Todinova S., Andreeva T., Langari A., Bogdanova D., Zlatareva E., Kalaydzhiev N., Milanov I., Taneva S.G. (2022). Platelets’ nanomechanics and morphology in neurodegenerative pathologies. Biomedicines.

[81] Wiest I., Wiemers T., Kraus M.-J., Neeb H., Strasser E.F., Hausner L., Frölich L., Bugert P. (2019). Multivariate platelet analysis differentiates between patients with alzheimer’s disease and healthy controls at first clinical diagnosis. J. Alzheimer’s Dis..

[82] Tirozzi A., Izzi B., Noro F., Marotta A., Gianfagna F., Hoylaerts M.F., Cerletti C., Donati M.B., de Gaetano G., Iacoviello L. (2020). Assessing genetic overlap between platelet parameters and neurodegenerative disorders. Front. Immunol..

[83] Koç E.R., Uzar E., Cirak Y., Demir Y.P., İlhan A. (2014). The increase of mean platelet volume in patients with alzheimer disease. Turk. J. Med. Sci..

[84] Palix C., Felisatti F., Gonneaud J., Kuhn E., Mézenge F., Landeau B., Chocat A., Quillard A., Egret S., Delarue M. (2022). Relationships between diabetes-related vascular risk factors and neurodegeneration biomarkers in healthy aging and alzheimer’s disease. Neurobiol. Aging.

[85] Merighi S., Battistello E., Casetta I., Gragnaniello D., Poloni T.E., Medici V., Cirrincione A., Varani K., Vincenzi F., Borea P.A. (2021). Upregulation of cortical a 2a adenosine receptors is reflected in platelets of patients with alzheimer’s disease. J. Alzheimer’s Dis..

[86] Oberacher H., Arnhard K., Linhart C., Diwo A., Marksteiner J., Humpel C. (2017). Targeted metabolomic analysis of soluble lysates from platelets of patients with mild cognitive impairment and alzheimer’s disease compared to healthy controls: Is pc aec40: 4 a promising diagnostic tool?. J. Alzheimer’s Dis..

[87] Wilkins H.M., Koppel S.J., Bothwell R., Mahnken J., Burns J.M., Swerdlow R.H. (2017). Platelet cytochrome oxidase and citrate synthase activities in apoe ε4 carrier and non-carrier alzheimer’s disease patients. Redox Biol..

[88] Kapoor A., Nation D.A., Alzheimer’s Disease Neuroimaging Initiative (2022). Platelet-derived growth factor-bb and white matter hyperintensity burden in apoe4 carriers. Cereb. Circ.-Cogn. Behav..

[89] Lee B.K., Kim M.H., Lee S.Y., Son S.J., Hong C.H., Jung Y.-S. (2020). Downregulated platelet mir-1233-5p in patients with alzheimer’s pathologic change with mild cognitive impairment is associated with aβ-induced platelet activation via p-selectin. J. Clin. Med..

[90] Gámez-Valero A., Campdelacreu J., Vilas D., Ispierto L., Gascón-Bayarri J., Reñé R., Álvarez R., Armengol M.P., Borràs F.E., Beyer K. (2021). Platelet mirna biosignature discriminates between dementia with lewy bodies and alzheimer’s disease. Biomedicines.

[91] Donovan L.E., Dammer E.B., Duong D.M., Hanfelt J.J., Levey A.I., Seyfried N.T., Lah J.J. (2013). Exploring the potential of the platelet membrane proteome as a source of peripheral biomarkers for alzheimer’s disease. Alzheimer’s Res. Ther..

[92] Yu H., Li M., Pan Q., Liu Y., Zhang Y., He T., Yang H., Xiao Y., Weng Y., Gao Y. (2022). Integrated analyses of brain and platelet omics reveal their common altered and driven molecules in alzheimer’s disease. MedComm.

[93] Milovanovic M., Eriksson K., Winblad B., Nilsson S., Lindahl T.L., Post C., Järemo P. (2014). Alzheimer and platelets: Low-density platelet populations reveal increased serotonin content in alzheimer type dementia. Clin. Biochem..

[94] Tajeddinn W., Fereshtehnejad S.-M., Ahmed M.S., Yoshitake T., Kehr J., Shahnaz T., Milovanovic M., Behbahani H., Höglund K., Winblad B. (2016). Association of platelet serotonin levels in alzheimer’s disease with clinical and cerebrospinal fluid markers. J. Alzheimer’s Dis..

[95] Veitinger M., Oehler R., Umlauf E., Baumgartner R., Schmidt G., Gerner C., Babeluk R., Attems J., Mitulovic G., Rappold E. (2014). A platelet protein biochip rapidly detects an alzheimer’s disease-specific phenotype. Acta Neuropathol..

[96] Reumiller C.M., Schmidt G.J., Dhrami I., Umlauf E., Rappold E., Zellner M. (2018). Gender-related increase of tropomyosin-1 abundance in platelets of alzheimer’s disease and mild cognitive impairment patients. J. Proteom..

[97] Zhao S., Zhao J., Zhang T., Guo C. (2016). Increased apoptosis in the platelets of patients with alzheimer’s disease and amnestic mild cognitive impairment. Clin. Neurol. Neurosurg..

[98] Zheng W., Fan D. (2022). Glucocerebrosidase mutations cause mitochondrial and lysosomal dysfunction in parkinson’s disease: Pathogenesis and therapeutic implications. Front. Aging Neurosci..

[99] Guzman-Martinez L., Maccioni R.B., Andrade V., Navarrete L.P., Pastor M.G., Ramos-Escobar N. (2019). Neuroinflammation as a common feature of neurodegenerative disorders. Front. Pharmacol..

[100] Bathina S., Das U.N. (2015). Brain-derived neurotrophic factor and its clinical implications. Arch. Med. Sci..

[101] Sidorova Y.A., Volcho K.P., Salakhutdinov N.F. (2019). Neuroregeneration in parkinson’s disease: From proteins to small molecules. Curr. Neuropharmacol..

[102] Duman R.S. (2004). Neural plasticity: Consequences of stress and actions of antidepressant treatment. Dialogues Clin. Neurosci..

[103] Zotey V., Andhale A., Shegekar T., Juganavar A. (2023). Adaptive neuroplasticity in brain injury recovery: Strategies and insights. Cureus.

[104] Zhang L., Shao Y., Tang C., Liu Z., Tang D., Hu C., Liang X., Hu Z., Luo G. (2022). Identification of novel biomarkers in platelets for diagnosing parkinson’s disease. Eur. Neurol..

[105] Montenegro P., Pueyo M., Lorenzo J.N., Villar-Martinez M.D., Alayón A., Carrillo F., Borges R. (2022). A secretory vesicle failure in parkinson’s disease occurs in human platelets. Ann. Neurol..

[106] Page M.J., Pretorius E. (2021). Platelet behavior contributes to neuropathologies: A focus on alzheimer’s and parkinson’s disease. Semin. Thromb. Hemost..

[107] Özdemir Z., Alagöz M.A., Bahçecioğlu Ö.F., Gök S. (2021). Monoamine oxidase-b (mao-b) inhibitors in the treatment of alzheimer’s and parkinson’s disease. Curr. Med. Chem..

[108] Jiang L., Zhong Z., Huang J., Bian H., Huang W. (2022). Monocytohigh-density lipoprotein ratio has a high predictive value for the diagnosis of multiple system atrophy and the differentiation from parkinson’s disease. Front. Aging Neurosci..

[109] Beura S.K., Yadav P., Panigrahi A.R., Singh S.K. (2023). Unveiling the mechanism of platelet dysfunction in parkinson’s disease: The effect of 6-hydroxydopamine on human blood platelets. Park. Relat. Disord..

[110] Bronstein J.M., Paul K., Yang L., Haas R.H., Shults C.W., Le T., Ritz B. (2015). Platelet mitochondrial activity and pesticide exposure in early parkinson’s disease. Mov. Disord..

[111] Delila L., Nebie O., Le N.T.N., Barro L., Chou M.L., Wu Y.W., Watanabe N., Takahara M., Buée L., Blum D. (2023). Neuroprotective activity of a virus-safe nanofiltered human platelet lysate depleted of extracellular vesicles in parkinson’s disease and traumatic brain injury models. Bioeng. Transl. Med..

[112] Tirozzi A., Parisi R., Cerletti C., Donati M.B., de Gaetano G., Iacoviello L., Gialluisi A. (2021). Genomic overlap between platelet parameters variability and age at onset of parkinson disease. Appl. Sci..

[113] Stanca I.-D., Criciotoiu O., Neamtu S.-D., Vasile R.-C., Berceanu-Bora N.-M., Minca T.-N., Pirici I., Rosu G.-C., Bondari S. (2022). The analysis of blood inflammation markers as prognostic factors in parkinson’s disease. Healthcare.

[114] Socha, Fife E., Kroc Ł., Kostka T. (2019). The association between platelet indices, cognitive screening tests and functional dependence screening questionnaires in hospitalized older people. Eur. Geriatr. Med..

[115] Albrecht D., Isenberg A.L., Stradford J., Monreal T., Sagare A., Pachicano M., Sweeney M., Toga A., Zlokovic B., Chui H. (2020). Associations between vascular function and tau pet are associated with global cognition and amyloid. J. Neurosci..

[116] Liang Q.-C., Jin D., Li Y., Wang R.-T. (2014). Mean platelet volume and platelet distribution width in vascular dementia and alzheimer’s disease. Platelets.

[117] Beard D.J., Brown L.S., Sutherland B.A. (2020). The rise of pericytes in neurovascular research. J. Cereb. Blood Flow Metab..

[118] Schröder S., Heck J., Groh A., Frieling H., Bleich S., Kahl K.G., Bosch J.J., Krichevsky B., Schulze-Westhoff M. (2022). White blood cell and platelet counts are not suitable as biomarkers in the differential diagnostics of dementia. Brain Sci..

[119] Donner L., Fälker K., Gremer L., Klinker S., Pagani G., Ljungberg L.U., Lothmann K., Rizzi F., Schaller M., Gohlke H. (2016). Platelets contribute to amyloid-β aggregation in cerebral vessels through integrin αiibβ3–induced outside-in signaling and clusterin release. Sci. Signal..

[120] Tayler H.M., MacLachlan R., Güzel Ö., Miners J.S., Love S. (2023). Elevated late-life blood pressure may maintain brain oxygenation and slow amyloid-β accumulation at the expense of cerebral vascular damage. Brain Commun..

[121] Samuel D., Bhat A.N., Prabhu V.M. (2020). Platelet indices as predictive markers of prognosis in critically ill patients: A prospective study. Indian J. Crit. Care Med. Peer-Rev. Off. Publ. Indian Soc. Crit. Care Med..

[122] Narasimhalu K., Ma L., De Silva D.A., Wong M.-C., Chang H.-M., Chen C. (2015). Elevated platelet-derived growth factor ab/bb is associated with a lower risk of recurrent vascular events in stroke patients. Int. J. Stroke.

[123] Bayat M., Zabihi S., Karbalaei N., Haghani M. (2020). Time-dependent effects of platelet-rich plasma on the memory and hippocampal synaptic plasticity impairment in vascular dementia induced by chronic cerebral hypoperfusion. Brain Res. Bull..

[124] Pan D., Rong X., Li H., Deng Z., Wang J., Liu X., He L., Xu Y., Tang Y. (2022). Anti-platelet therapy is associated with lower risk of dementia in patients with cerebral small vessel disease. Front. Aging Neurosci..

[125] Fourier A., Escal J., Bernard E., Lachman I., Perret-Liaudet A., Leblanc P., Quadrio I. (2019). Development of an automated capillary nano-immunoassay—Simple western assay—To quantify total tdp43 protein in human platelet samples. Anal. Bioanal. Chem..

[126] McCully S.P., Schreiber M.A. (2013). Traumatic brain injury and its effect on coagulopathy. Semin. Thromb. Hemost..

[127] Zhang J., Zhang F., Dong J.-F. (2018). Coagulopathy induced by traumatic brain injury: Systemic manifestation of a localized injury. Blood J. Am. Soc. Hematol..

[128] Jehan F., Zeeshan M., Kulvatunyou N., Khan M., O’Keeffe T., Tang A., Gries L., Joseph B. (2019). Is there a need for platelet transfusion after traumatic brain injury in patients on p2y12 inhibitors?. J. Surg. Res..

[129] Davis P.K., Musunuru H., Walsh M., Cassady R., Yount R., Losiniecki A., Moore E.E., Wohlauer M.V., Howard J., Ploplis V.A. (2013). Platelet dysfunction is an early marker for traumatic brain injury-induced coagulopathy. Neurocritical Care.

[130] Raz L., Knoefel J., Bhaskar K. (2016). The neuropathology and cerebrovascular mechanisms of dementia. J. Cereb. Blood Flow Metab..

[131] Nekludov M., Bellander B.-M., Blombäck M., Wallen H.N. (2007). Platelet dysfunction in patients with severe traumatic brain injury. J. Neurotrauma.

[132] El-Menyar A., Al-Thani H., Mansour M.F. (2023). Dementia and traumatic brain injuries: Underestimated bidirectional disorder. Front. Neurol..

[133] Nebie O., Carvalho K., Barro L., Delila L., Faivre E., Renn T.-Y., Chou M.-L., Wu Y.-W., Nyam-Erdene A., Chou S.-Y. (2021). Human platelet lysate biotherapy for traumatic brain injury: Preclinical assessment. Brain.

[134] Kalelioglu T., Yuruyen M., Gultekin G., Yavuzer H., Ozturk Y., Kurt M., Topcu Y., Doventas A., Emul M. (2017). The neutrophil and platelet to lymphocyte ratios in people with subjective, mild cognitive impairment and early alzheimer’s disease. Eur. Psychiatry.

[135] Budson A.E., Solomon P.R. (2012). New criteria for alzheimer’s disease and mild cognitive impairment: Implications for the practicing clinician. Neurologist.

[136] Yin C., Li S., Zhao W., Feng J. (2013). Brain imaging of mild cognitive impairment and alzheimer’s disease. Neural Regen. Res..

[137] Vega J.N., Newhouse P.A. (2014). Mild cognitive impairment: Diagnosis, longitudinal course, and emerging treatments. Curr. Psychiatry Rep..

[138] Vatanabe I.P., Pedroso R.V., Manzine P.R., Chagas M.H.N., de Morais Fabrício D., Grigoli M.M., Naves M.A., Pott H., Cominetti M.R. (2021). Adam10: Biomarker of mild cognitive impairment but not of cognitive frailty. Exp. Gerontol..

[139] Bermejo-Bescós P., Martín-Aragón S., Jiménez-Aliaga K., Benedí J., Felici E., Gil P., Ribera J.M., Villar Á.M. (2013). Processing of the platelet amyloid precursor protein in the mild cognitive impairment (mci). Neurochem. Res..

[140] McGuinness B., Fuchs M., Barrett S.L., Passmore A.P., Johnston J.A. (2016). Platelet membrane β-secretase activity in mild cognitive impairment and conversion to dementia: A longitudinal study. J. Alzheimer’s Dis..

[141] Wang R.-T., Jin D., Li Y., Liang Q.-C. (2013). Decreased mean platelet volume and platelet distribution width are associated with mild cognitive impairment and alzheimer’s disease. J. Psychiatr. Res..

[142] Zhang Y., Liu J., Wei Z., Mei J., Li Q., Zhen X., Zhang Y. (2023). Elevated serum platelet count inhibits the effects of brain functional changes on cognitive function in patients with mild cognitive impairment: A resting-state functional magnetic resonance imaging study. Front. Aging Neurosci..

[143] Balietti M., Giuli C., Fattoretti P., Fabbietti P., Postacchini D., Conti F. (2016). Cognitive stimulation modulates platelet total phospholipases a 2 activity in subjects with mild cognitive impairment. J. Alzheimer’s Dis..

[144] Gattaz W.F., Talib L.L., Schaeffer E.L., Diniz B.S., Forlenza O.V. (2014). Low platelet ipla 2 activity predicts conversion from mild cognitive impairment to alzheimer’s disease: A 4-year follow-up study. J. Neural Transm..

[145] Saito S., Kojima S., Oishi N., Kakuta R., Maki T., Yasuno F., Nagatsuka K., Yamamoto H., Fukuyama H., Fukushima M. (2016). A multicenter, randomized, placebo-controlled trial for cilostazol in patients with mild cognitive impairment: The comcid study protocol. Alzheimer’s Dement. Transl. Res. Clin. Interv..

[146] Yu H., Liu Y., He T., Zhang Y., He J., Li M., Jiang B., Gao Y., Chen C., Ke D. (2021). Platelet biomarkers identifying mild cognitive impairment in type 2 diabetes patients. Aging Cell.

[147] Nelligan M., Nellis M.E., Mauer E.A., Gerber L.M., Traube C. (2023). Association between platelet transfusion and delirium in critically ill children. Children.

[148] Pasqui E., de Donato G., Brancaccio B., Casilli G., Ferrante G., Cappelli A., Palasciano G. (2022). The predictive role of inflammatory biochemical markers in post-operative delirium after vascular surgery procedures. Vasc. Health Risk Manag..

[149] Şaşkın H., Özcan K.S., Yildirim S. (2022). The role of inflammatory parameters in the prediction of postoperative delirium in patients undergoing coronary artery bypass grafting. Cardiovasc. J. Afr..

[150] Rudiger A., Begdeda H., Babic D., Krüger B., Seifert B., Schubert M., Spahn D.R., Bettex D. (2016). Intra-operative events during cardiac surgery are risk factors for the development of delirium in the icu. Crit. Care.

[151] Mitsuru I., Takeshita Y., Kawaguchi M. (2020). Preoperative serum biomarkers in the prediction of postoperative delirium following abdominal surgery. Geriatr. Gerontol. Int..

[152] Lechowicz K., Szylińska A., Listewnik M., Drożdżal S., Tomska N., Rotter I., Kotfis K. (2021). Cardiac delirium index for predicting the occurrence of postoperative delirium in adult patients after coronary artery bypass grafting. Clin. Interv. Aging.

[153] Oyama T. (2022). Higher Neutrophil-to-Lymphocyte Ratio, Mean Platelet Volume, and Platelet Distribution Width are Associated with Postoperative Delirium in Patients Undergoing Esophagectomy: A Retrospective Observational Study.

[154] Li X., Wang G., He Y., Wang Z., Zhang M. (2022). White-cell derived inflammatory biomarkers in prediction of postoperative delirium in elderly patients undergoing surgery for lower limb fracture under non-general anaesthesia. Clin. Interv. Aging.

[155] Jiang X., Shen Y., Fang Q., Zhang W., Cheng X. (2020). Platelet-to-lymphocyte ratio as a predictive index for delirium in critically ill patients: A retrospective observational study. Medicine.

[156] Lu W., Lin S., Wang C., Jin P., Bian J. (2023). The potential value of systemic inflammation response index on delirium after hip arthroplasty surgery in older patients: A retrospective study. Int. J. Gen. Med..

[157] Soler-Sanchis A., Martínez-Arnau F.M., Sánchez-Frutos J., Pérez-Ros P. (2022). Challenges in the detection of clinically useful biomarkers for the diagnosis of delirium in older people in the emergency department—A case–control study. Life.

[158] Thisayakorn P., Tangwongchai S., Tantavisut S., Thipakorn Y., Sukhanonsawat S., Wongwarawipat T., Sirivichayakul S., Maes M. (2021). Immune, blood cell, and blood gas biomarkers of delirium in elderly individuals with hip fracture surgery. Dement. Geriatr. Cogn. Disord..

[159] Yang Y., Liu Y., Xu R., Jiao Y., Hao J., Sun Y., Gu X., Zhang W. (2023). The predictive values of platelet mitochondrial mass and quantity during the perioperative period in elderly patients on the occurrence of postoperative delirium. Zhonghua Yi Xue Za Zhi.

[160] Silva A.C., Almeida S., Laço M., Duarte A.I., Domingues J., Oliveira C.R., Januário C., Rego A.C. (2013). Mitochondrial respiratory chain complex activity and bioenergetic alterations in human platelets derived from pre-symptomatic and symptomatic huntington’s disease carriers. Mitochondrion.

[161] Ehinger J.K., Morota S., Hansson M.J., Paul G., Elmér E. (2016). Mitochondrial respiratory function in peripheral blood cells from huntington’s disease patients. Mov. Disord. Clin. Pract..

[162] Denis H.L., Lamontagne-Proulx J., St-Amour I., Mason S.L., Rowley J.W., Cloutier N., Tremblay M.-È., Vincent A.T., Gould P.V., Chouinard S. (2019). Platelet abnormalities in huntington’s disease. J. Neurol. Neurosurg. Psychiatry.

[163] Carrizzo A., Di Pardo A., Maglione V., Damato A., Amico E., Formisano L., Vecchione C., Squitieri F. (2014). Nitric oxide dysregulation in platelets from patients with advanced huntington disease. PLoS ONE.

